# The HECT ubiquitin‐protein ligases UPL1 and UPL2 are involved in degradation of *Arabidopsis thaliana* ACC synthase 7

**DOI:** 10.1111/ppl.70030

**Published:** 2025-01-06

**Authors:** Małgorzata Marczak, Agata Cieśla, Maciej Janicki, Syed Muhammad Muntazir Mehdi, Piotr Kubiak, Agnieszka Ludwików

**Affiliations:** ^1^ Preclinical Development Department, Celon Pharma S.A., Research & Development Centre Poland; ^2^ Laboratory of Biotechnology, Institute of Molecular Biology and Biotechnology, Faculty of Biology Adam Mickiewicz University in Poznań Poznań Poland; ^3^ Institute of Biology, Biotechnology and Environmental Protection, Faculty of Natural Sciences University of Silesia in Katowice Katowice Poland; ^4^ Department of Biotechnology and Food Microbiology University of Life Sciences Poznań Poland

## Abstract

Ethylene is an important plant hormone whose production relies on the action of key enzymes, one of which is 1‐aminocyclopropane‐1‐carboxylate synthase (ACS). There are three classes of ACS, which are all partially regulated by degradation through the ubiquitin‐proteasome system (UPS), which regulates ethylene production. Arabidopsis has a single class III ACS, ACS7, but although it is known to be degraded by the 26S proteasome, the UPS proteins involved are poorly characterised. In this work, we used mass spectrometry to identify novel components of the ubiquitin system that may contribute to the regulation of ethylene biosynthesis via ACS7. We found two HECT‐type ligases, UPL1 and UPL2, which regulate ACS7 stability. In vitro experiments showed that UPL1 and UPL2 E3 ligases directly control ACS7 turnover. In addition, increased ethylene levels were observed in UPL1‐ and UPL2‐knockout plants in response to NaCl and NaCl+MG132 treatment, respectively. Under the same conditions, we observed increased ACS7 transcript levels in *upl1* compared to WT plants under control and stress conditions, further confirming that UPL1 and UPL2 regulate ACS7‐dependent ethylene production in response to stress. We used molecular modelling to predict ACS7 ubiquitylation sites and cell‐free degradation assays to verify that lysine residues at positions 174, 238 and 384 regulate ACS7 protein stability. Overall, this study provides new insights into the regulation of ACS7 protein stability, and hence ethylene production, in plant growth and development and the response to stress.

## 1 INTRODUCTION

The ubiquitin‐proteasome system (UPS) dynamically controls protein abundance to maintain cellular homeostasis. In plants, the UPS controls a range of cellular processes such as growth, development and stress responses by regulating the synthesis, action and crosstalk of hormones. The plant hormone ethylene is highly dependent on the ubiquitin system (Wang et al. 2004; Christians et al. 2009; Cai et al. 2018; Shan et al. 2020). In recent years, key molecular players linking the UPS to the ethylene‐dependent signalling, ethylene biosynthesis or response pathways have been investigated but are not yet fully understood. Ethylene regulates growth, development and senescence and cooperates with other plant hormones to trigger specific plant responses under normal and stress conditions (Khan et al. 2024). In general, ethylene is considered to be a growth‐suppressing hormone (Dubois et al. 2018). In roots, ethylene regulates primary root growth by reducing the size and cell number of the root meristem. This phenotype is suppressed by ethylene‐insensitive or ethylene‐resistant mutations (Thomann et al. 2009). In leaves of plants submitted to stress, the resulting increased ethylene levels appear to have a negative effect on the cell cycle and a positive effect on cell expansion (Marsch‐Martinez et al. 2006; Fatma et al. 2022). Overproduction of ethylene leads to dwarf phenotypes (Vogel et al. 1998; Dubois et al. 2018; Khan et al. 2024). In the ein2 mutant with reduced ethylene sensitivity, leaf size is increased as a result of increased cell expansion (Feng et al. 2015). On the other hand, reduced ethylene biosynthesis has a significant effect on crop yield. Zea mays lines with reduced ethylene biosynthesis and sensitivity show a significant increase in grain yield when exposed to drought stress in the field (Habben et al. 2014). Alternative approaches, such as altering the expression of ethylene response and signalling genes, have been used successfully in the field but have resulted in unwanted pleiotropic phenotypes (Dubois et al. 2018). Therefore, the identification of new mechanisms and players regulating ethylene production is crucial for a better understanding of plant productivity under stress conditions.

A key step in ethylene biosynthesis is catalysed by 1‐aminocyclopropane 1‐carboxylate synthase (ACC synthase, ACS) (Wang et al. 2002). In Arabidopsis, the ACC synthases are encoded by a multigene family of 12 members, although only eight genes encode functional ACSs (Yamagami et al. 2003; de Poel and Van Der Straeten 2014). Based on their C‐terminal domain, three types of ACS can be distinguished. Type I isoforms (ACS1, ACS2 and ACS6) contain the longest C‐terminal domain, which accommodates phosphorylation sites for calcium‐dependent protein kinases (CDK) and mitogen‐activated protein kinases (MAPK). The other two ACS types have significantly reduced C‐terminal domains. Type II isozymes (ACS4, ACS5, ACS8, ACS9, ACS11) include CDPK sites only, while type III ACS (ACS7) lacks any kinase‐targeted domain (Tatsuki and Mori 2001; Yamagami et al. 2003; Liu and Zhang 2004; Sebastià et al. 2004; Lyzenga et al. 2012).

The consensus kinase‐targeting sequences present at the type‐I ACS C‐terminus suggest extensive phosphorylation of the protein. As might be expected, a subset of MAP kinases has been identified that modify ACSs together with protein phosphatases; thus, MPK3, MPK6, CPK4 and CPK11 are known to phosphorylate type I ACSs (Liu and Zhang 2004; Luo et al. 2014). Arabidopsis casein kinase 1 (CK1.8) was found to phosphorylate ACS5 (Tan and Xue 2014). The type III protein ACS7, which does not contain C‐terminal motifs, can be phosphorylated by CPK16 (Huang et al. 2013). Protein phosphatases that dephosphorylate ACS isoforms have also been identified; for example, PP2A and ABI1 PP2C can dephosphorylate ACS6 (Skottke et al. 2011; Ludwikow et al. 2014), while ABI1/ABI2/HAB1 dephosphorylate ACS7 (Marczak et al. 2020). It is evident that, in addition to reversible phosphorylation, other post‐translational modifications, including ubiquitination, have a significant impact on the ACSs, leading to changes in ethylene production (Lyzenga and Stone 2012; Ludwików 2015). It is now recognized that ACS stability is regulated by the interplay between phosphorylation and ubiquitination, independent of the presence of the C‐terminal domain.

Ubiquitination, a conserved regulatory pathway for protein degradation, plays an important role in plant signalling. Protein degradation via the ubiquitin (Ub) system occurs in two steps. The first step is a covalent attachment of ubiquitin to the internal lysine of a substrate, while the second is the degradation of the tagged target protein via the 26S proteasome (Ciechanover and Schwartz 1998). The target protein can be modified by a single moiety of ubiquitin or by a branched polyubiquitin chain (Lyzenga et al. 2012). The conjugation of ubiquitin to a lysine residue of the target substrate requires three sequential enzyme reactions involving ubiquitin‐activating enzyme (E1), ubiquitin‐conjugating enzymes (UBCs; E2 – also known as ubiquitin‐carrier proteins) and ubiquitin ligases (E3), which transfer Ub from E2 to a lysine residue of the target protein (Pao et al. 2018). Current evidence suggests that E3 enzymes bring specificity to the ubiquitination in different tissues, at various developmental stages, and under a range of environmental conditions (Chen and Hellmann 2013). In the ethylene biosynthesis pathway only a small number of E3s have been recognized: ETO1, CUL3‐RING (CRL3) and ETO1‐like (EOL1 and EOL2) ligases mediate type II ACS degradation (Wang et al. 2004; Yoshida et al. 2005), while the RING‐type E3 ligase XBAT32 is responsible for ACS4/7 degradation in Arabidopsis (Prasad et al. 2010; Lyzenga et al. 2012). However, no E3s have yet been recognised for type I ACSs.

In plants, E3 ubiquitin ligases are classified into four groups based on their characteristic domains: HECT (Homologous to E6‐associated protein C‐Terminus), RING (Really Interesting New Gene, also called Ubiquitin Protein Ligases, UPLs), U‐Box, and CRL (Cullin‐RING Ligases) (Vierstra 2009; Shu and Yang 2017; Zhou and Zeng 2017). The RING E3s contain a cysteine‐rich domain that coordinates two zinc atoms and have been shown to play key roles in germination, development, signalling (including ABA signalling) and responses to abiotic stress. U‐box E3 ligases are multidomain proteins with a highly conserved E2 docking module that controls numerous developmental and stress‐response processes. CRLs, which are divided into SCF, BTB, DDB and APC subtypes, are involved in phytohormone signalling pathways, plant development and various abiotic stress responses. Finally, the HECT E3s all have the same three‐domain structure: the N‐terminal is different in each family member but is followed in all cases by a specific domain for recognition and conjugation of the target protein, and finally, the C‐terminal HECT domain with a cysteine residue at the active site (Bernassola et al. 2008; Qian et al. 2020).

UPL1 and UPL2 are HECT‐type E3 ligases that were first identified (Bates and Vierstra 1999) during the screening of EST libraries to identify and define the functions of HECT E3s in plants. Sequence analysis revealed them to be encoded by two exceptionally long genes (genomic sequence >13 kb; CDS >11 kb) that are approximately 95% identical to each other. The UPL1 and UPL2 proteins are among the largest proteins in Arabidopsis, with a molecular mass of approximately 405 kDa. The significant sequence similarity between UPL1 and UPL2 is most likely an effect of gene duplication (Bates and Vierstra 1999). Further studies have identified other members of the E3 HECT family, which has been divided into five subfamilies in *Arabidopsis thaliana*: UPL1/UPL2 (subfamily V), UPL3/UPL4 (subfamily I), UPL5 (subfamily VI), UPL6 (subfamily III) and UPL7 (subfamily II) (Downes et al. 2003; Marín 2013). This protein family plays an important role in development, maturation, leaf senescence and seed production (Miao and Zentgraf 2010; Lan and Miao 2019). UPLs target plant hormone‐specific transcription activators (TAs) such as NPR1 and EIN3, salicylic acid‐ and ethylene‐responsive TAs, respectively, for proteasomal degradation (Wang et al. 2022). An important role of the UPL family in plant hormone signalling has been suggested but is not fully understood. In this study, we uncover how the HECT ligases UPL1 and UPL2 regulate the ethylene biosynthetic pathway by controlling the turnover of ACS7, the type III ACC synthase.

## 2 MATERIALS AND METHODS

### 2.1 Plant materials, growth conditions and treatments

*Arabidopsis thaliana* seeds, ecotype Col‐0 wild type, *upl1* (Salk_063972C; the European Arabidopsis Stock Centre, NASC) and *upl2* (Salk_151696C; the European Arabidopsis Stock Centre, NASC) were used. Seeds were surface‐sterilized with 30% bleach and 70% ethanol. After treatment at 4°C for 2 days, seeds were germinated and grown in soil or Murashige and Skoog medium (containing 0.8% agar and 2% saccharose). For cell‐free degradation assays, 7‐day‐old seedlings were treated with 100 μM MG132 (Sigma‐Aldrich).

### 2.2 Vector construction

ACS7 sequences were PCR‐amplified and introduced into the entry vector, pENTR/SD/D‐TOPO (Invitrogen). Lysine residues in positions 86, 174, 238, and 435 of the ACS7 protein were changed to arginine by mutating the corresponding DNA sequence using the QuikChange II XL Site‐Directed Mutagenesis Kit (Agilent), according to the manufacturer's protocol. Each mutated variant was confirmed by sequencing. pENTR constructs were obtained using the Gateway system. For bacterial expression, ACS7 and mutants of ACS7 were recombined into the pDEST15 vector to obtain GST‐tagged fusion proteins. All the clones were confirmed by restriction digestion and sequencing. Primers used are listed in Table 2.

### 2.3 RNA extraction and RT–qPCR analysis

RNA isolation and cDNA synthesis were performed as previously described (Ludwików et al. 2009). Total RNA was isolated using an RNAeasy kit following the manufacturer's instructions (Qiagen). cDNA was synthesized using the Maxima first‐strand cDNA synthesis kit with DNAse (Thermo Fisher Scientific) according to the manufacturer's instructions. Total RNA (2–3 μg) was treated with DNase (included in the Maxima first‐strand cDNA synthesis kit), and then first‐strand cDNA was synthesised using half of the DNase‐treated RNA, with the remaining DNase‐treated RNA used as a negative control. The manufacturer's instructions for the SYBR Green Real‐Time PCR Master Mix reagent (Thermo Fisher Scientific) were followed, and RT‐PCR was performed on an Applied Biosystems QuantStudio™ 7 Flex Real‐Time PCR System (Applied Biosystems) in a volume of 20 μL. Three biological replicates were performed in triplicate for each cDNA sample, negative control and no‐template control. ACT2 was used as a reference. Data analyses were performed using QuantStudio Software (Applied Biosystems). After obtaining data values from the qPCR machine, the 2^ΔΔCt^ method was used to calculate relative expression levels (Livak and Schmittgen 2001) and all gene expression levels were normalized against ACT2. Primers used in the qPCR assay are listed in Table 2.

### 2.4 Expression and purification of proteins

Recombinant GST–ACS7 and GST‐fusion versions of the ACS7 mutants (K86R, K174R, K238R, K384R) were expressed in *Escherichia coli* strain BL21‐CodonPlus (DE3)‐RIL. A single colony was inoculated into LB medium supplemented with ampicillin (100 mg L^−1^) and incubated at 37°C until a final OD_600_ of ~0.8 was reached. Protein expression was induced using isopropyl β‐D‐1‐thiogalactopyranoside (IPTG) at a final concentration of 0.5 mM followed by 4 h cultivation at 37°C. Cells were harvested by centrifugation, resuspended in lysis buffer (1x PBS pH 7.4, 150 mM NaCl, 1% Triton X‐100, 1 mM PMSF) and disrupted by sonication on ice. The recombinant GST fusion proteins were purified by affinity chromatography using Glutathione‐Sepharose 4B (GE Healthcare) according to the manufacturer's protocols.

### 2.5 ACS7 protein complex purification

ACS7‐GFP‐His was overexpressed in mesophyll protoplasts from 3‐week‐old WT Col‐0 Arabidopsis plants. Protoplast isolation was performed as described in Ludwikow et al. 2014 and Mituła et al. 2015. Immunoprecipitation of ACS7‐GFP‐His was performed using GFP‐trap agarose (Chromtek™) according to the manufacturer's recommendations. Briefly, for one purification reaction, approximately 3600 μL portions of pelleted protoplasts expressing ACS7‐GFP‐His or WT protoplasts (as a control) were resuspended in 500 μL lysis buffer (10 mM Tris–HCl pH 7.5, 150 mM NaCl, 0.5% Nonidet™ P40, protease inhibitor cOmplete™ protease inhibitor cocktail Roche) and disrupted for 2 min, 1000 oscillations per minute, using TissueLyser II (Qiagen). After protein extraction, samples were spun down at high speed (15 min/25 000 g/4°C), and the soluble protein fraction was combined with 50 μL GFP‐trap agarose. Immunoprecipitation was performed overnight at 4°C with slow rotation on a Stuart Tube Rotator (Stuart). After incubation, the samples were centrifuged to sediment the GFP‐Trap Agarose, the supernatant was removed, and the resin was washed three times (1 mL wash buffer, 5 min with rotation) with wash buffer: 10 mM Tris–HCl pH 7.5, 150 mM NaCl, 0.05% Nonidet™ P40. Samples were prepared in triplicate. Immunoprecipitated proteins were stored on GFP‐trap agarose and sent for a mass spectrometry analysis.

### 2.6 Identification of ACS7 protein complexes by LS‐MS/MS

Mass spectrometry analyses were performed in the Mass Spectrometry Laboratory IBB PAS, Pawińskiego 5A, 02–106 Warsaw. LC–MS/MS was performed on an LTQ‐Orbitrap Velos Pro (ThermoFisher Scientific) coupled to a U3000 RSLCnano HPLC (ThermoFisher Scientific). To generate the list of identified proteins, database searches were performed using Mascot (Matrix Science, London, UK; v.2.5.0) Ion Search against the TAIR10 pep_20101214 database (35386 sequences; 14482855 residues) and the cRAP database (http://www.thegpm.org/cRAP/) as a common contamination db. Mascot search parameters: and assuming trypsin digestion and 2 missed cleavages, fixed modifications: Methylation (C), variable modifications: GlyGly (K), oxidation (M). Mass value: monoisotopic, protein mass tolerance: +/− 5 ppm. Mascot was searched with a fragment ion mass tolerance of 0.80 Da and a parent ion tolerance of 15 ppm. To determine non‐specific interactions with the GFP trap resin, all proteins identified in the control samples were removed from the list of identified proteins. The resulting list of proteins from each sample was then searched for E3 ubiquitin ligases.

### 2.7 Cell‐free degradation assay

The cell‐free degradation assay was performed as described (Ludwikow et al. 2014; Mitula et al. 2015; Marczak et al. 2020). Seven‐day‐old WT Col‐0, *upl1* and *upl2* seedlings grown in ½ MS media were treated with proteasome inhibitor MG132. Dimethyl sulfoxide (DMSO; the solvent for MG132) was used as a control. Then, 300–500 μg total protein extract was mixed with recombinant protein (GST‐ACS7 WT, K86R, K174R, K238R or K384R). Samples were incubated at 22°C in the light and collected at the indicated time points. GST–ACS7 protein was detected with anti‐GST (1:5000 or 1:8000; MoBiTec). Ponceau staining was used to check for equal loading.

### 2.8 Immunoblotting

Denatured proteins were separated on a 10% SDS–PAGE gel and transferred to Immobilon P (Merck Millipore) or Immun‐Blot® PVDF membrane (Bio‐Rad). Membranes were blocked for 1 h in PBS containing 0.01% or 0.05% Tween 20 (PBS‐T, pH 7.4) and 5% (w/v) skimmed milk. Membranes were washed three times before incubation with a primary rabbit antibody against the GST tag (1:5000; MoBiTec), followed by an HRP‐conjugated secondary antibody (1:25000, Agrisera, AS09 602). After three washes with PBS‐T, blots were developed using standard ECL (ThermoFisher Scientific) according to the manufacturer's instructions. Blots were visualised using the ChemiDoc imaging system (Bio‐Rad).

### 2.9 Computational prediction of key lysine residues for ubiquitination using structural bioinformatics

For structure prediction and preparation, models of Ub and ACS7 were generated using AlphaFold2 (Jumper et al. 2021). ACS7 is reported to form a dimer, and consequently, the multimer option was selected during modelling with AF2. The highest‐ranked structure was used for further analysis. The structures obtained were prepared using the Preparation Wizard protocol implemented in Schrodinger Suite 2022 and minimised in OPLS4 using the Prime module. The ACS7 structure obtained from AF2 was used to predict the conformation of loops that were absent in the 7dlw structure (derived from crystallography) in the pdb database. The resulting complete structure of ACS7 was prepared and minimised, and Ub was used as input during protein–protein docking studies.

For protein–protein docking, the Cluspro web server was used following the blind docking protocol (Kozakov et al. 2017). No constraints were applied during protein–protein docking. The resulting complex models were ranked according to a balanced scheme. In the next step, the Haddock web server (Van Zundert et al. 2016; Honorato et al. 2021) was used, and calculations were performed using additional constraints and default settings. Specifically, the single lysine of the target protein and G76 of Ub were selected as active residues during the macromolecular docking protocol. The results obtained were ranked based on the Haddock scoring function, the distance between residue K of ACS7 and G76 of Ub, and the binding energy calculated by the Molecular Mechanics Generalised Born Surface Area (MM‐GBSA) method using the Prime module (Jacobson et al. 2004). The binding energy value was determined as the difference between the energy of the ACS7‐Ub complex and the energy of the unbound ACS7 and Ub. In the MM‐GBSA binding energy estimation, the entropy term was neglected and the variable‐dielectric generalised Born solvation model (Li et al. 2011) was applied in the OPLS4 force field (Lu et al. 2021) with a minimised sampling method. The evaluated structures were analysed and visualised using PyMOL (DeLano 2002).

Molecular dynamics simulations were performed using the Desmond software package (Desmond, 2022). A Nose‐Hoover thermostat and a Martyna‐Tobias‐Klein barostat were used in the NPT ensemble class simulation of each system, maintaining a temperature of 300 K and a constant pressure of 1 bar. The integration time step was set to 2 fs and the total simulation time was 100 ns. Hydrogen‐heavy atomic bonds were kept rigid using the SHAKE algorithm. Long‐range Coulomb interactions were treated with a smooth‐particle Ewald mesh, while short‐range Coulomb interactions had a cutoff of 9 Å. Structures were prepared and minimised prior to simulation. Using the TIP3P water model, each system was solvated in an orthorhombic box after charges were balanced and 0.15 M NaCl was added. OPLS4 force field parameters were applied to each simulation. Post‐processing analysis, including clustering, RMSD and RMSF calculations, was performed using Desmond tools.

### 2.10 Ethylene measurements

Ethylene measurements were performed on 2‐week‐old seedlings of WT Col‐0, *upl1* and *upl2* mutant plants grown in 25 mL GC vials. In each vial, 120 seeds were placed on 8 mL solid ½ MS medium and sealed with a vial cap. Seeds were stratified at 4°C in the dark for 2 days, then transferred to the growth chamber and grown for 14 days. Twenty‐four hours before treatment, the vials were opened, and the accumulated gases were removed by pipetting. Plants were treated with 120 μL of either 100 μM MG132, 150 mM NaCl or 100 μM MG132 plus 150 mM NaCl. The vials were resealed. Ethylene was quantified by gas chromatography as described (Marczak et al. 2020).

## 3 RESULTS

### 3.1 Identification of E3 ligases UPL1 and UPL2 in the ACS7 protein complex

To identify novel components of the ubiquitin system that may contribute to the regulation of ethylene biosynthesis, an ACS7‐GFP‐His construct was expressed in wild‐type Arabidopsis protoplasts treated with the proteasome 26S inhibitor MG132. Protein samples isolated from non‐transformed protoplasts were used as a negative control for non‐specific binding to the beads. Purified proteins from the samples were identified by mass spectrometry. After the elimination of non‐specific interactions, we generated a list of proteins specifically identified in affinity‐purified ACS7‐GFP complexes (Table S1), and these proteins were classified using Gene Ontology (GO) (Table S2). Classification based on cellular process revealed a group of proteins related to components of the ubiquitin system (Table 1). Among them, we recognised RPN1A 26S proteasome regulatory subunit S21A (AT2G20580.1); 20S proteasome alpha subunit PAD1 (AT3G51260.1); 26S proteasome regulatory complex, non‐ATPase subcomplex, Rpn2/Psmd1 subunit (AT1G04810.1); proteasome component (PCI) domain proteins (AT5G42970. 1, AT5G14250.1, AT4G19006.1); the ubiquitin‐specific proteases UBP4 (AT2G22310.1), UBP6 (AT1G51710.1) and UBP14 (AT3G20630.1); and the ubiquitin carboxyl‐terminal hydrolase UCH2 (AT1G65650.1) – all of which are involved in the deubiquitylation process. We also identified enzymes of the ubiquitination pathway represented by the E1 ubiquitin‐activating enzyme UBA1 (AT2G30110.1). Of particular interest were proteins classified as E3 ligases UPL1 (AT1G55860), UPL2 (AT1G70320), HOS1 (AT2G39810) and SKP1 (AT1G75950), which generally exhibit high substrate specificity and are involved in many cellular regulatory mechanisms. Therefore, we chose UPL1 and UPL2 to further confirm their putative role in the regulation of the ethylene biosynthetic pathway.

**TABLE 1:** Proteins from ubiquitin‐proteasome pathway identified in ACS7 protein complexes.

Accession	Protein description	Mass	Mascot Score	emPAI	Number of peptides	Rank	Peptide sequence
**Ubiquitin E3 ligases**							
**AT1G55860.1**	UPL1 | ubiquitin‐protein ligase 1 | chr1:20879900–20895393 REVERSE LENGTH = 3930	434872	99	0.01	2	1	K.TEVTDYELKPGGR.N
						1	R.DLGGLDDTIFR.L
**AT1G75950.1**	SKP1, ASK1, ATSKP1, SKP1A, UIP1 | S phase kinase‐associated protein 1 | chr1:28516715–28517454 FORWARD LENGTH = 160	17984	95	0.26	1	1	K.AEAVEGAATSDDDLK.A
**AT2G39810.1**	HOS1 | ubiquitin‐protein ligases | chr2:16612941–16617802 FORWARD LENGTH = 927	106267	52	0.04	1	1	K.GSVSPQLQDVQTLR.E
**AT1G70320.1**	UPL2 | ubiquitin‐protein ligase 2 | chr1:26488745–26501281 REVERSE LENGTH = 3658	405664	49	0.01	1	1	K.TEVTDYELKPGGR.N
**Proteasome components**							
**AT2G20580.1**	RPN1A, ATRPN1A | 26S proteasome regulatory subunit S2 1A | chr2:8859211–8864699 FORWARD LENGTH = 891	98634	230	0.19	4	1	R.MLQYGEQNIR.R
							K.QESVEATAEVSK.T
							R.VGQAVDVVGQAGRPK.T
							R.IGAIMGLGISYAGSQNDQIR.N
**AT2G30110.1**	ATUBA1, MOS5, UBA1 | ubiquitin‐activating enzyme 1 | chr2:12852632–12857369 REVERSE LENGTH = 1080	121003	213	0.19	5	1	K.LLNFKPLR.E
							K.STVAASAAAVINPR.F
							K.LQDLNNAVVVSSLTK.S
							K.LISIATAINTGQGDLK.V
							K.AVLNPMAAMFGGIVGQEVVK.A
**AT3G02200.2**	| Proteasome component (PCI) domain protein | chr3:406692–408919 FORWARD LENGTH = 417	47031	189	0.43	4	1	R.AVIEFVK.A
							R.LLSLVDLASDESGK.I
							K.MDQMNQVLIVSR.S
							K.YLATFSNEDAQVLDEAKEEAVR.A
**AT5G42970.1**	COP8, FUS4, EMB134, COP14, CSN4, FUS8, ATS4 | Proteasome component (PCI) domain protein | chr5:17237470–17240649 REVERSE LENGTH = 397	45159	127	0.32	3	1	K.FLEAALR.Y
							K.EIAQFTLTQIQPR.V
							K.LILSSVLSSNDLLQAQR.F
**AT3G51260.1**	PAD1 | 20S proteasome alpha subunit PAD1 | chr3:19031086–19032746 FORWARD LENGTH = 250	27412	68	0.17	1	1	R.ALLEVVESGGK.N
**AT5G14250.1**	COP13, CSN3, FUS11 | Proteasome component (PCI) domain protein | chr5:4597970–4600561 FORWARD LENGTH = 429	48079	59	0.19	2	1	K.IGELEALVVAR.N
							R.NAEFEEDKNLGLVK.Q
**AT4G19006.1**	| Proteasome component (PCI) domain protein | chr4:10409349–10411347 REVERSE LENGTH = 386	44131	49	0.1	1	1	R.TIPLSVIAER.T

### 3.2 UPL1 and UPL2 E3 ligases regulate ACS7 stability

**FIGURE 1:**
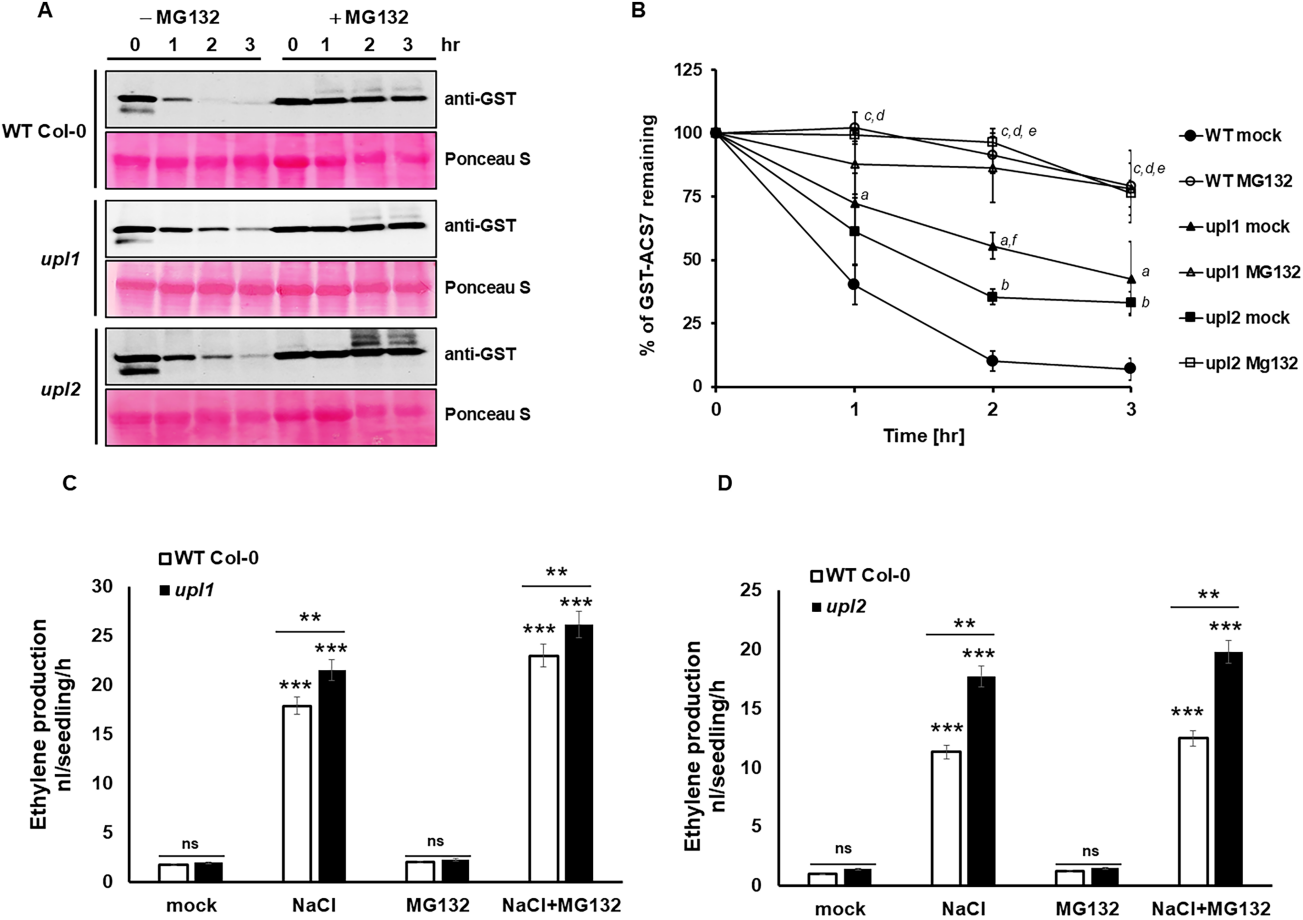
UPL1 and UPL2 E3 ligases regulate the stability of ACS7. (A) GST‐ACS7 (55 kDa) was incubated with *upl1*, *upl2* and WT Col‐0 extracts with or without MG132 for the indicated times. Western blotting was performed using anti‐GST antibodies. Ponceau staining was used to assess loading. (B) Half‐life plot for cell‐free degradation of GST‐ACS7. ImageJ software was used to quantify signals and normalise to the mock control. Data are averaged from three replicates with SD indicated. (C‐D) Ethylene production in response to MG132. Seedlings of *upl1*, *upl2* and WT Col‐0 lines were treated with MG132 in GC vials and headspace ethylene was measured 24 h later. Stars indicate statistically significant changes at **p < 0.01, *** *p* < 0,001 as indicated by Student's t‐test. ns indicate *p* > 0.05. Data are the mean of three replicates ±SD.

UPL1 and UPL2 belong to subfamily V of the HECT Ub E3 ligases and have very similar domain compositions, suggesting that they have overlapping functions. As UPL1 and UPL2 are large proteins (3681 aa and 3658 aa, respectively), we were unable to generate full‐length UPL constructs for analysis. Instead, to evaluate the contribution of UPL1 and UPL2 to the degradation of ACS7 protein, we studied *upl1* and *upl2* T‐DNA insertion lines. After confirming homozygous insertion lines for both loci (Supplemental Figure 1), we performed a cell‐free degradation assay (Figure 1). For this purpose, recombinant GST‐ACS7 protein was incubated with protein extracts prepared from WT Col‐0 and *upl1*‐ and *upl2*‐knockout lines. On protein gel immunoblots, we observed a significant delay in GST‐ACS7 degradation in protein extracts from *upl1* (T_1/2_ ~ 3 h) and *upl2* (T_1/2_ ~ 1.5 h) lines compared to WT Col‐0 (T_1/2_ ~ 1 h) (Figure 1A‐B). In addition, GST‐ACS7 degradation was significantly reduced in protein extracts treated with MG132, documenting the role of UPL1 and UPL2 in regulating ACS7 stability.

To further test whether UPLs contribute to ethylene production and regulation of ACS7 stability, we analysed ethylene levels in *upl1*, *upl2* and WT Col‐0 seedlings (Figure 1). Interestingly, in response to MG132 treatment, *upl1* and *upl2* showed comparable ethylene levels to WT Col‐0 plants (Figure 1C‐D). We hypothesised, therefore, that UPLs may regulate ACS7 stability under certain conditions, such as in response to stress. Previous data show that ACS7 is induced in response to abiotic stresses, including salt (Xiong et al. 2014). Therefore, we determined ethylene production in *upl1*, *upl2* and WT Col‐0 seedlings in response to 150 mM NaCl treatment. As expected, ethylene production was significantly higher in salt‐treated *upl* lines than in the WT (Figure 1C‐D). The increase in ethylene production in both *UPL* knockout lines was significantly more profound when treated simultaneously with NaCl and MG132 than the WT. There was no significant difference in ethylene production between WT and *upl* lines under normal conditions and in response to MG132 treatment. These results demonstrate that UPL1 and UPL2 E3 ligases regulate ACS7 stability in response to salt stress.

**FIGURE 2:**
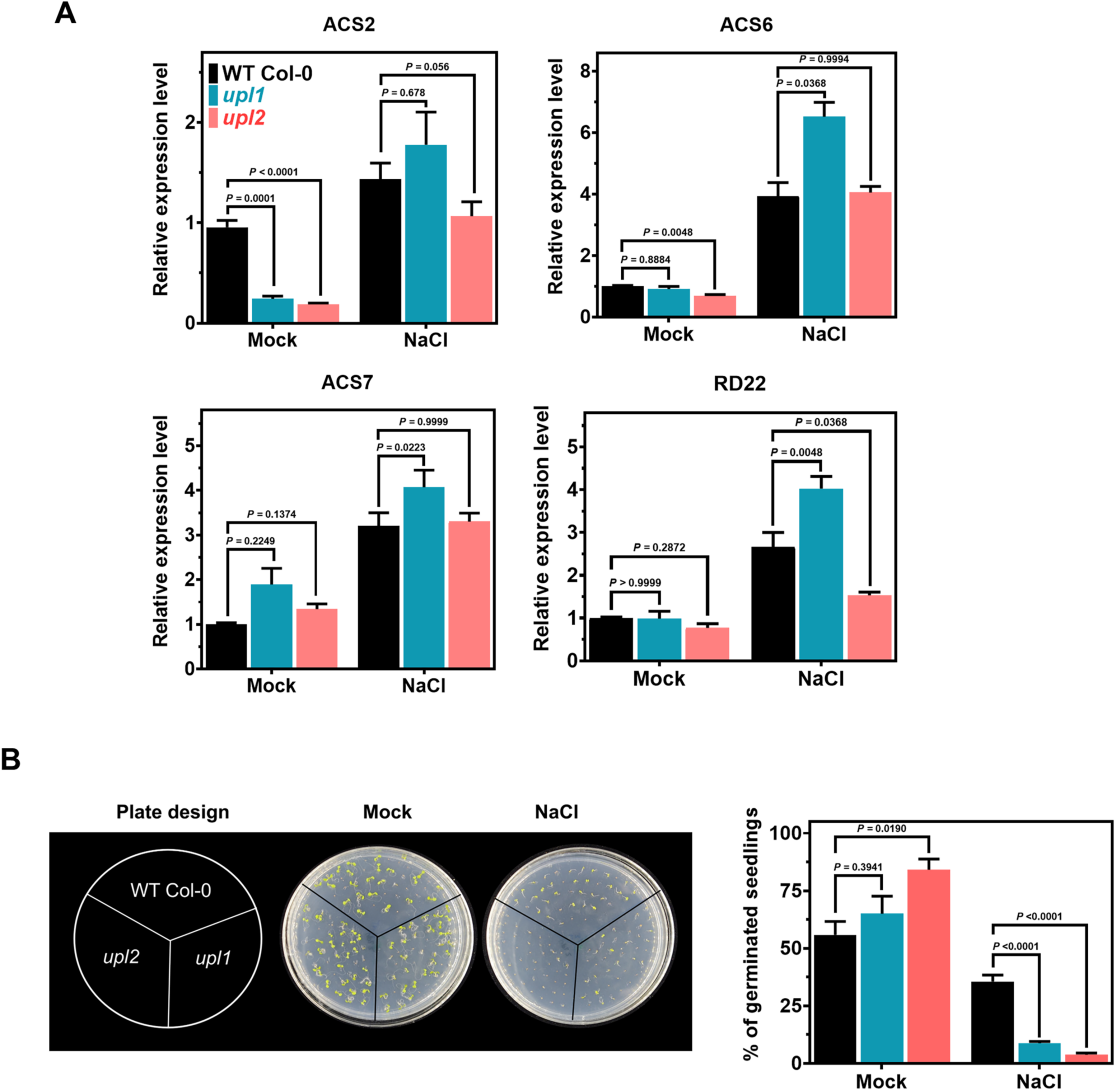
Salt stress response in WT Col‐0, *upl1‐5 and upl2* lines. (A) Vernalized seeds of wild type (Col‐0), *upl1‐5* and *upl2* lines were germinated on MS medium for two weeks and treated with or without (Mock) 150 mM NaCl for six hours, respectively, then RNA was extracted and transcript levels of the *ACS2*, *ACS6* and *ACS7* genes were analyzed by qRT‐PCR, with *ACT2* gene as an internal control. The *RD22* gene was used as a positive control in the WT Col‐0, *upl1‐5 and upl2* lines to check for induction by NaCl. Values are standard errors of the mean (*n* = 3) biological replicates with independent technical triplicates for each and data were analysed by two‐way ANOVA with Tukey's HSD for significance; (B) The WT Col‐0, *upl1‐5 and upl2* lines were germinated on MS medium with or without 150 mM NaCl. Percentages of cotyledon greening were recorded 5 days after sowing. At least 40 seeds per genotype were used in each replicate in three independent experiments. Values are the mean ± SD of three replicates. Data were analysed by two‐way ANOVA with Tukey's HSD for significance.

We next examined the expression pattern of *ACS7* and two other stress‐associated ACS isoforms, *ACS2* and *ACS6*, in NaCl‐treated *upl1* and *upl2* lines (Figure 2A), as ethylene production during a stress response is also regulated at the transcriptional level. As a marker for stress treatment, we used the *RD22* transcript, which is up‐regulated by salt and drought. Under normal growth conditions, ACS2 has a lower expression level in the *upl1* and *upl2* background than the WT. We also observed a significant decrease in *ACS6* transcript abundance in *upl2* compared to WT Col‐0. Upon NaCl treatment, we observed a significant decrease in *ACS2* transcript levels in the *upl2* line and an increase in *ACS6* and *ACS7* transcript abundance in the *upl1*‐knockout line compared to WT Col‐0. It is interesting to note that the salt‐induced *RD22* transcript levels are significantly reduced in the *upl2* background compared to the WT and *upl1* backgrounds. In summary, the knockout of UPL1 and UPL2 resulted in increased ethylene production in response to salt treatment. This increased ethylene evolution is associated with increased *ACS7* transcript levels in the *upl1* but not in the *upl2* line. These data, therefore suggest that UPL1 and UPL2 play an important role in ethylene evolution in response to NaCl.

### 3.3 Prediction of ACS7 ubiquitination sites using molecular modelling

**FIGURE 3:**
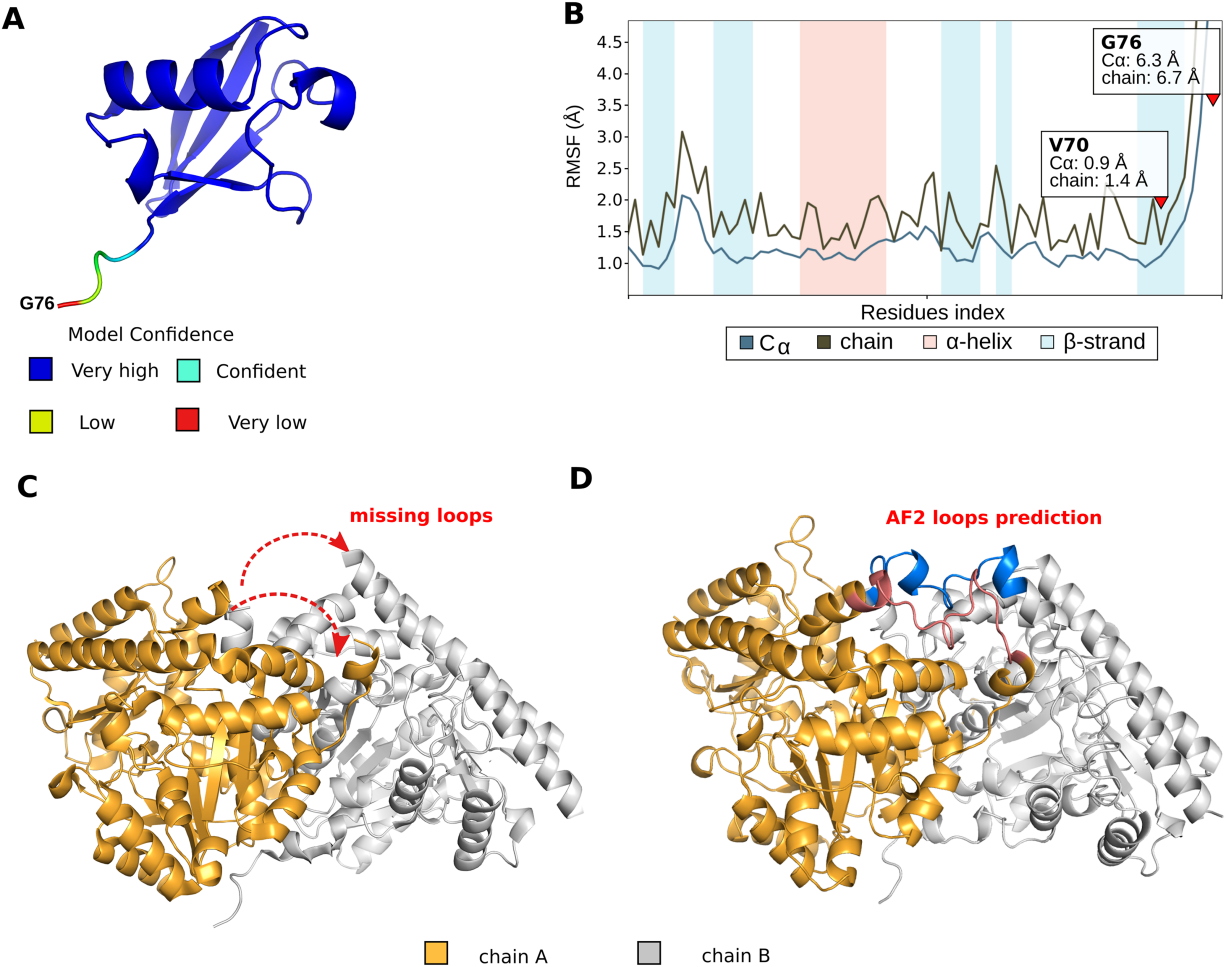
Structure of Ub and ACS7. (A) Prediction of Ub structure using AF2 with highlighted model confidence score. (B) RMSF diagram representing positional fluctuations of all amino acids for the Ub model based on the MD data after postprocessing using Desmond package; RMSF is the root mean square fluctuation calculated for the trace Cɑ or side chain of a given residue (chain). The RMSF value is shown for V70 and G76 residues. (C) Structure of ACS7 homodimer (pdb code: 7dlw), with missing loop regions labelled. (D) Full structure of ACS7 homodimer, including the two loops missing from 7dlw. Protein loop conformation was predicted using AF2 code and manually added to the 7dlw structure (shown as red and blue). The full AF2 prediction for ACS7 homodimer is shown in supplementary data.

**FIGURE 4:**
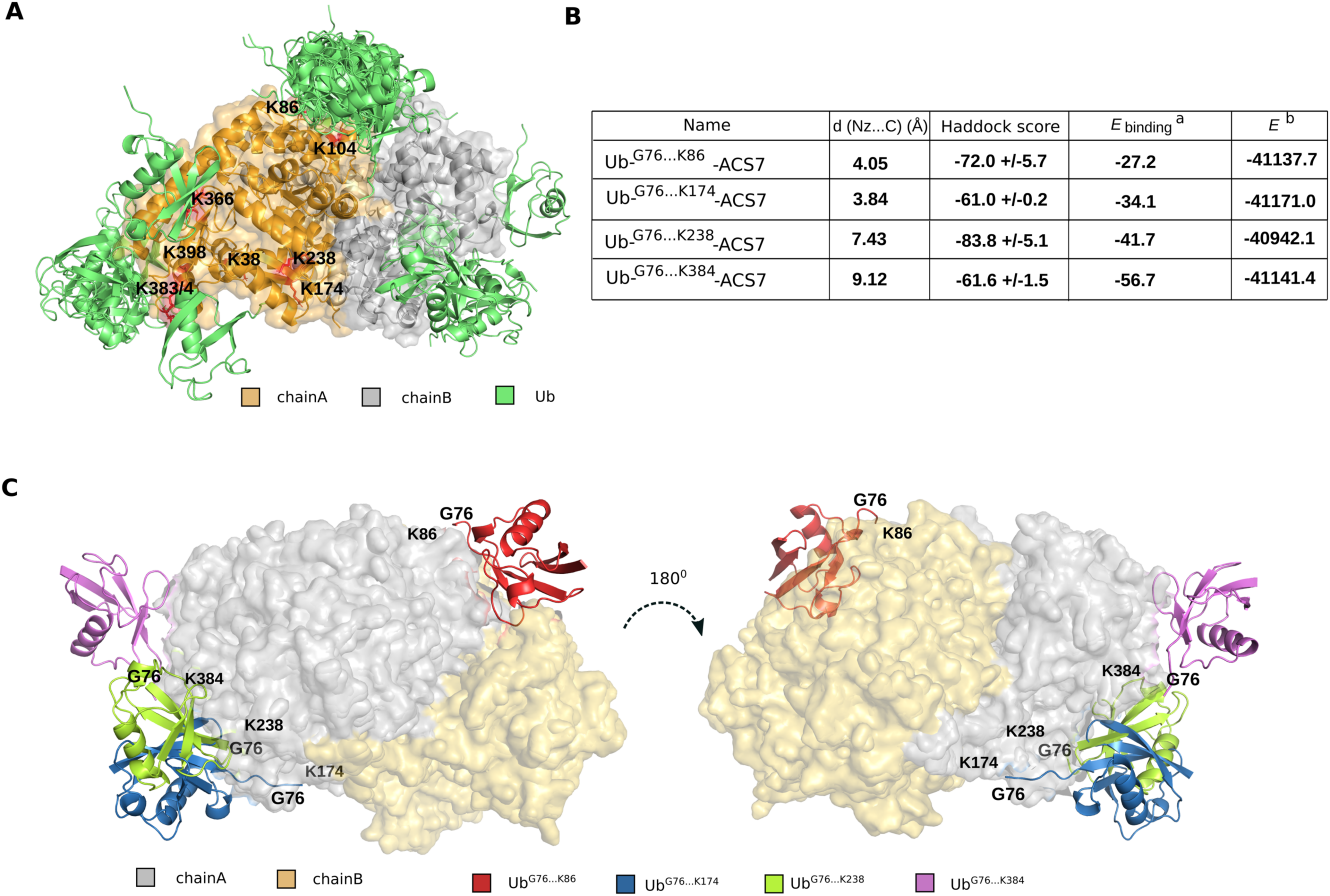
Structural prediction of ubiquitination sites in the ACS7 homodimer. (A) Initial results from the Cluspro webserver blind docking step with pre‐selected K residues labelled as potential ubiquitination sites. (B) The table summarises the best models of ACC7‐Ub complexes, including the distance in Å between the nitrogen atom (Nz) of the MAPKK18 lysine residue and the alpha carbon atom of the ubiquitin G76 residue, the Haddock score; a) estimate of MM‐GBSA binding energy (kcal/mol) obtained from the Prime module; b) energy (kcal/mol) of minimised ACC7‐Ub structure obtained from the Prime module. (C) Best models of the ACC7‐Ub complex with chain A and chain B of the ACC7 protein marked and the best binding pose of the Ub molecule chosen in each case.

To identify the key lysine residues in ACS7 that can be ubiquitinated, we analyzed the structural capabilities of potential ubiquitination sites using a molecular modelling approach, which included macromolecular docking and MD simulation. Ubiquitination is a post‐translational modification in which ubiquitin attaches to the target protein via a covalent bond between the Cα atom of the carboxyl group of the glycine residue (G76) of Ub and the nitrogen atom of a lysine amino group in the target protein. To construct models of ACS7‐Ub, the model of Ub developed using AlphaFold2 (Jumper et al. 2021) and the structure of ACS7 obtained from X‐ray crystallography (pdb code: 7dlw) (Xu et al. 2021) were used. The Ub model has an excellent confidence score, except for the C‐terminal part, which appears to be a disordered region. MD data confirmed that residues forming the C‐tail of Ub have a high RMSF value (Figure 3A‐B). The structure of ACS7 (pdb code: 7dwl) has regions missing from residues 73 to 90 for chain A and from residues 77 to 90 for chain B (Figure 3C‐D), again likely due to disorder. We modelled this region by manually connecting the missing loops obtained from the AF2 model of ACS7 (Figures S1‐S3) to the crystallographic structure of 7dlw. The structures of Ub and ACS7 obtained were prepared using the wizard tool and minimised in the OPLS4 force field (Lu et al. 2021) the resulting structures were used in subsequent analysis (Figure 4).

ACS7 has twenty lysine residues, one of which, K285, was not accessible for Ub binding and participates in PPG ligand binding according to structure 7dlw. In addition, K435, the last lysine at the C‐terminus of the ACS7 structure, was not involved in proteasomal degradation. These two lysine residues were thus ignored in our in‐silico approach. To determine which lysine residues of ACS7 can be modified by ubiquitin attachment, we used a two‐step approach (Tajdel‐Zielińska et al. 2023) involving (1) blind docking using Cluspro (Kozakov et al. 2017) and (2) docking with residues defined as active using Haddock software (Van Zundert et al. 2016; Honorato et al. 2021). From the first step, nine lysine residues (K86, K238, K174, K384, K366, K104, K398, K383, K38) were selected as potential binding sites (Figure 4 A‐C), and these amino acids, together with G76 of Ub, were used as active residues in the second step of the docking protocol. In this step, fourteen conformers of Ub obtained from MD simulation data were used, with the C‐terminal region designed to remain flexible during the docking process. The evaluated ACS7‐Ub complexes were ranked according to Haddock score, binding energy, and distance between the ACS7 lysine residue and G76 of Ub. Based on their ranking, K86, K174, K238 and K384 were selected from the initial list of nine for experimental validation as potentially ubiquitinated lysine residues in ACS7.

### 3.4 Lysine residues at positions 174, 238 and 384 regulate ACS7 protein stability

**FIGURE 5:**
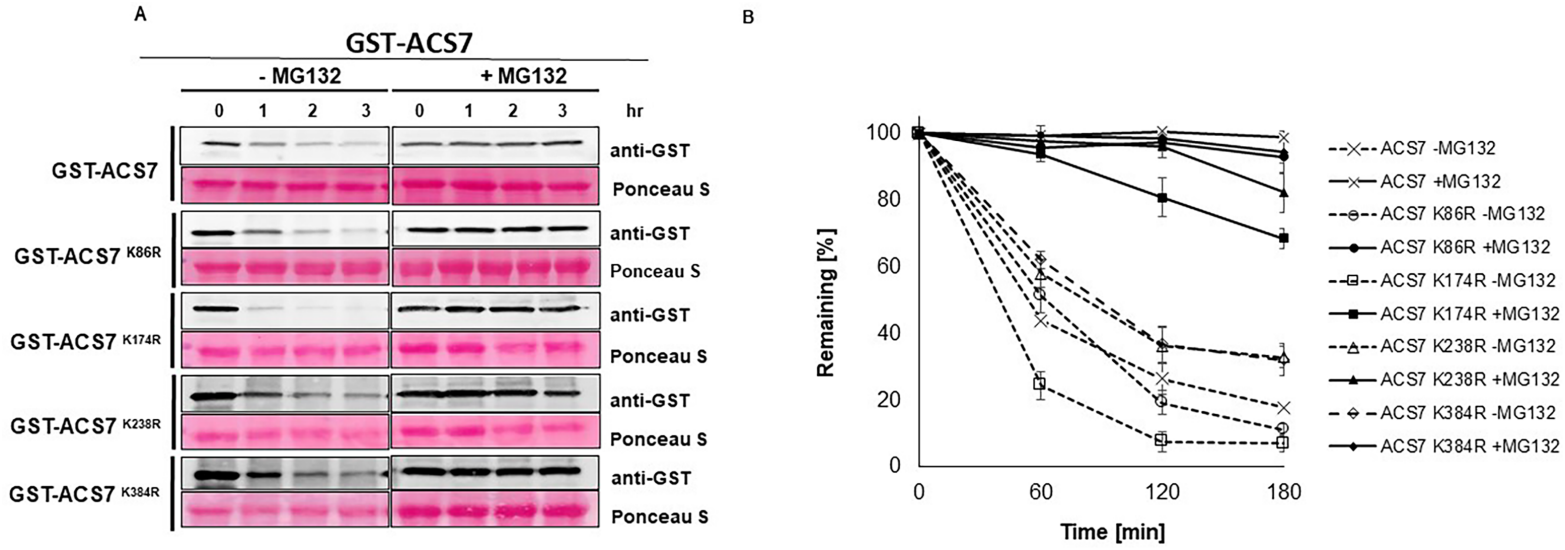
Cell‐free assays for the degradation of ACS7 and mutated variants of ACS7 in wild‐type plant extracts with or without MG132 treatment. (A) GST‐ACS7 (55 kDa) and mutants were incubated with WT Col‐0 extracts for the indicated times and analysed by western blotting using anti‐GST antibodies. Ponceau staining was used to assess loading. (B) Half‐life plot for cell‐free degradation of GST‐ACS7 and the K86R, K174R and K384R mutants of GST‐ACS7. Signals were quantified using ImageJ software and normalized to the control (mock). The data represent average values from three replicates with SD indicated.

Modelling experiments identified a list of potential ACS7 lysine residues to which ubiquitin may be attached (Table 2): lysine residues at positions 86,174, 238 and 384 are surface exposed and can interact with the ubiquitin molecule. Replacement of lysine with arginine blocks ubiquitin binding. Therefore, to confirm our prediction, lysine‐to‐arginine mutants (K86R, K174R, K238R and K384R) for the above residues were generated, the corresponding mutant GST‐ACS7 constructs were expressed in a bacterial expression system, and the recombinant proteins were used in a cell‐free degradation assay (Figure 5). The degradation half‐life of two mutants, K238R and K384R, was about 90 min, which is significantly longer than GST‐ACS7 in the WT plant extract (about 60 min). In contrast, the half‐life of K174R was shorter than that of wild‐type ACS7 (about 30 min) in WT Col‐0 plant extracts. The half‐lives of mutant and non‐mutant ACS7 proteins in WT Col‐0 plant extracts treated with MG132, the 26S proteasome inhibitor, were longer than 180 min. The data strongly suggest that lysine at positions 238 and 384 are required for ACS7 protein stability. These results indicate that lysine residues K238 and K384 are critical for ACS7 protein turnover.

**TABLE 2:** Primers used in the study. The obtained top scored ACS7‐UBQ complex according to the distance in Å between nitrogen atom (Nz) from ACS7 lysine residue and alfa carbon atom (Cα) from ubiquitine G76 residue, haddock score which was assessed on the basics of macromolecular docking simulation and energy values obtained during geometry optimization of each ACS7‐UBQ complex using Prime module (energy*) and Macromodel module (energy**) (Schrodinger suite 2022). Each ACS7‐UBQ complex was named based on the number of the corresponding ACS7 lysine residue that was used as docking constraints, the ubiquitin conformation obtained from the MD analysis, and the cluster number obtained from the Haddock postprocessing analysis.

ACS7‐UBQ complex name	d(Nz…Cα)	Haddock score	Energy*	Energy **
**ACS7_K384_U11_cluster2**	**3.87**	**−77.8**	**−41200.4**	**−203526.562**
**ACS7_K366_U10_cluster7**	**3.79**	**−65.3**	**−41177.7**	**−203456.875**
**ACS7_K359_U_6_cluster9**	**3.51**	**−60.5**	**−41144.4**	**−203426.156**
**ACS7_K366_U10_cluster4**	**3.83**	**−77**	**−41182.6**	**−203418.312**
**ACS7_K225_U_6_cluster4**	**3.82**	**−67.7**	**−41127.8**	**−203383.859**
**ACS7_K86_U11_cluster1**	**3.99**	**−97.2**	**−41191.5**	**−203379.25**
**ACS7_K435_U4_cluster1**	**3.76**	**−60.6**	**−41194.9**	**−203371.219**
**ACS7_K174_U10_cluster3**	**3.81**	**−71.9**	**−41152.5**	**−203371.125**
**ACS7_K121_U11_cluster3**	**3.65**	**−68.6**	**−41118.9**	**−203351.031**
**ACS7_K104_U_9_cluster9**	**3.8**	**−60.3**	**−41148.3**	**−203323.188**
**ACS7_K238_U2_cluster2**	**3.87**	**−60.8**	**−41166.5**	**−203313.812**
**ACS7_K398_U_7_cluster3**	**3.64**	**−67**	**−41118.6**	**−203297.875**
**ACS7_K435_U5_cluster1**	**3.49**	**−75.3**	**−41181.1**	**−203284.062**
**ACS7_K121_U7_cluster2**	**3.94**	**−83.2**	**−41171**	**−203245.984**
**ACS7_K366_U4_cluster1**	**3.96**	**−69.7**	**−41175.6**	**−203245.062**
**ACS7_K435_U7_cluster1**	**3.75**	**−81.2**	**−41088.2**	**−203243.547**
**ACS7_K366_U5_cluster8**	**3.96**	**−72.6**	**−41161.2**	**−203236.188**
**ACS7_K174_U7_cluster5**	**3.98**	**−61**	**−41166**	**−203232.859**
**ACS7_K435_U11_cluster1**	**3.9**	**−65.2**	**−41141.4**	**−203231.016**
**ACS7_K383_U12_cluster2**	**3.86**	**−67.6**	**−41116.5**	**−203228.797**
**ACS7_K384_U12_cluster3**	**3.86**	**−62.8**	**−41153.5**	**−203227.188**
**ACS7_K86_U6_cluster3**	**3.94**	**−66.2**	**−41144.8**	**−203214.406**
**ACS7_K38_U10_cluster3**	**3.72**	**−73.2**	**−41176.5**	**−203199.188**
**ACS7_K358_U_6cluster1**	**3.89**	**−72.3**	**−41087.3**	**−203197.891**
**ACS7_K226_U3_cluster1**	**3.44**	**−61**	**−41164.2**	**−203179.516**
**ACS7_K225_U_6_cluster1**	**3.48**	**−62.9**	**−41145.3**	**−203168.172**
**ACS7_K104_U_10_cluster4**	**3.88**	**−89.9**	**−41097.7**	**−203163.703**
**ACS7_K337_U_12_cluster6**	**3.52**	**−70.8**	**−41163.3**	**−203160.453**
**ACS7_K75_U6_cluster2**	**3.9**	**−69**	**−41129.6**	**−203142.938**
**ACS7_K104_U_8_cluster13**	**3.66**	**−64**	**−41144.4**	**−203132.156**
**ACS7_K104_U_5_cluster1**	**3.6**	**−65.9**	**−41113.9**	**−203127.875**
**ACS7_K337_U_7_cluster6**	**3.61**	**−60.7**	**−41103**	**−203110.828**
**A337_U_10_cluster1**	**3.91**	**−90.1**	**−41141.6**	**−203106.438**
**A435_U3_cluster3**	**3.48**	**−62.7**	**−41131.2**	**−203101.578**
**A38_U11_cluster3**	**3.8**	**−68**	**−41110**	**−203098.641**
**A384_U4_cluster4**	**3.85**	**−78.5**	**−41094.5**	**−203081.672**
**A104_U_5_cluster4**	**3.78**	**−65.1**	**−41097.8**	**−203034.781**
**A174_U12_cluster_10**	**3.87**	**−67.2**	**−41109.9**	**−203162.453**
**A174_U12_cluster_3**	**3.93**	**−64.4**	**−41054.5**	**−203226.406**
**A86_U11_cluster7**	**3.69**	**−69.6**	**−41058**	**−203300.109**

## 4 DISCUSSION

Proteasomal degradation plays an important role in the regulation of ACS proteins, resulting in altered ethylene biosynthesis. On the other hand, ethylene is elevated during development and stress responses, so it is crucial to tightly regulate its production. Key enzymes in ethylene synthesis are the ACC synthases, which in Arabidopsis comprise a large gene family divided into three classes (Hyun and Kieber 2005). Arabidopsis class III ACS has a single member, ACS7, whose function and regulation remain unclear. The lack of regulatory sequences within the C‐terminal tail of Arabidopsis ACS7 has raised questions about how it responds to abiotic or biotic stress. Recent studies have clearly shown that ACS7 is degraded by the 26S proteasome (Lyzenga et al. 2012; Marczak et al. 2020), but the UPS proteins involved are poorly characterised, except for XBAT32, a RING‐type E3 ligase that regulates the stability of both ACS7 and ACS4 proteins (Lyzenga et al. 2012). Here, we show that ACS7 is also regulated by the E3 ligases UPL1 and UPL2 in response to NaCl treatment. In addition, we used molecular modelling to predict ACS7 ubiquitination sites and verified that lysine residues at positions 174, 238 and 384 regulate ACS7 protein stability.

Mass spectrometry is a powerful method for identifying the composition of protein complexes. We used this method to identify the UPS machinery involved in the regulation of ACS7, including the E3 ubiquitin ligases. We identified two HECT‐type ligases, UPL1 and UPL2, that regulate the stability of ACS7. Interestingly, the mechanism by which HECT ligases promote substrate turnover is beginning to emerge. It is suggested that HECT ligases may contribute to protein turnover by targeting proteasome regulatory subunits for degradation, by delivering ubiquitinated substrates to the proteasome or by elongating existing ubiquitin chains (Wang and Spoel 2022). Our in vitro experiments clearly demonstrate that UPL1 and UPL2 E3 ligases directly control ACS7 turnover (Figure 1A‐B). As a result, we observed increased ethylene levels in *upl1* and *upl2* knockout plants in response to NaCl and NaCl+MG132 treatment, respectively (Figure 1C‐D). In addition, we observed increased ACS7 transcript levels in *upl1* compared to WT plants under control and stress conditions (Figure 2), suggesting that UPL1 and UPL2 regulate ACS7‐dependent ethylene production during the response to stress.

To gain further insight into the mechanism of ubiquitination of ACS7, we modelled the structure of the ACS7‐Ub complex and analysed residues in ACS7 that are potentially important for the interaction between these proteins. We selected the lysine residues at positions 86, 174, 238 and 384 in the primary structure of ACS7 to analyse their effect on its ubiquitination and subsequent degradation. Replacement of the lysine residues with arginine at positions 238 and 384 slowed down ACS7 degradation, suggesting that these are the critical sites for ACS7 proteasome‐dependent protein turnover. Remarkably, the ACS7 K174R variant exhibited a shorter half‐life than the non‐mutated protein in the cell‐free degradation assay. A similar effect was observed when ACS7 lysine 435 was replaced by arginine (Lyzenga et al. 2012). We hypothesise that these two lysine residues, K174 and K435, are more likely to be used for other post‐translational modifications (e.g. acetylation, methylation, SUMOylation) (Wang and Cole 2020) than ubiquitination and thus may still affect ACS7 stability. As we have shown previously (Marczak et al. 2020), the regulation of ACS7 requires a complex interplay between post‐translational modifications. Given that ACS7 can form functional heterodimers with other ACSs (Tsuchisaka and Theologis 2004; Tsuchisaka et al. 2009), juggling different types of post‐translational modification alongside the formation of various heterodimers will affect the stability of ACSs, which in turn will modulate ethylene production levels.

The identification of ubiquitination sites has greatly increased our understanding of ubiquitinated proteins and the UPS‐dependent cellular mechanisms that operate in the cell. Arabidopsis ACS7 has been implicated in vascular development (Yang et al. 2020), leaf senescence (Sun et al. 2017), and root gravitropism (Huang et al. 2013). It also regulates tolerance to salt, osmotic and heat stresses (Dong et al. 2011). From this perspective, ubiquitination of ACS7 has implications for plant development and stress responses (Vanderstraeten et al. 2019; Park et al. 2021). In the future, a more detailed knowledge of how the UPS influences the stability/modification of ACS protein regulation may have a significant contribution to the development of agronomic traits. The ubiquitin system has been reported to be involved in the control of plant agronomic traits in rice and maize (Varshney and Majee 2022).

## 5 CONCLUSION

**FIGURE 6:**
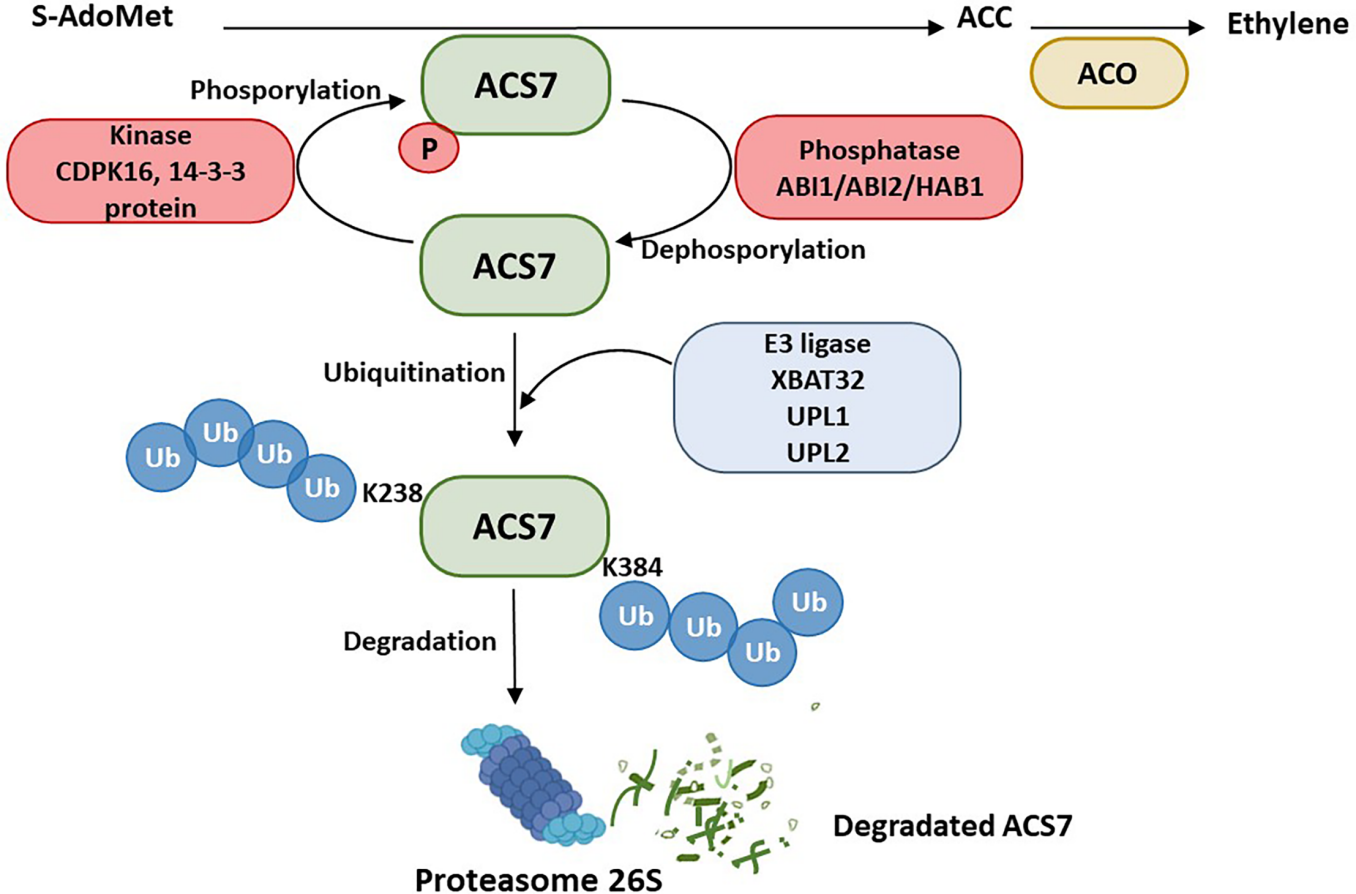
A model for the regulation of ACS7 protein stability. The stability of ACS7 is regulated by a complex network of reversible phosphorylation events and proteasomal degradation. Phosphorylation of ACS7 by kinases (CDPK16 and 14–3‐3 protein) leads to activation of ACS7 and an increase in ethylene biosynthesis. Protein phosphatases 2C, ABI1, ABI2 and HAB1, which are important components of the abscisic acid signalling pathway, interact with ACS7. Dephosphorylation of ACS7 reduces its stability. UPL1, UPL2 and XBAT32 act as E3 ligases that recognise ACS7 and add ubiquitin, targeting the protein for proteasomal degradation. Lysine residues critical for ubiquitination are located at positions 238 and 384 in the amino acid sequence of ACS7.

The current study provides new information on the mechanism of ACS7 protein stability and the regulation of ethylene production in plants. The role of posttranslational modifications in determining type III ACS protein stability has been partially defined. Previous data suggested that serine residues S48 and S85 play a critical role in ACS7 degradation (Marczak et al. 2020). C‐terminally truncated ACS7 is efficiently ubiquitinated, suggesting that ubiquitination and phosphorylation/dephosphorylation sites are located outside the C‐terminal domain. These mutations can impair ACS7 ubiquitination and inhibit proteasomal degradation. Together, the available data allow us to propose a model for the mechanism of ACS7 protein degradation (Figure 6). Continued research into the turnover of ACS proteins will provide further insights into ethylene production and its role in plant growth, development and the response to environmental stress.

## AUTHOR CONTRIBUTIONS

M.M., A.C. and M.J performed the main part of the experiments, analyzed and interpreted the data, drafted and revised the manuscript; P.K. and S.M.M.M. performed experiments, analyzed and interpreted the data, revised the manuscript; A.L. designed the study, analyzed and interpreted the data, drafted and revised the manuscript.

## FUNDING INFORMATION

This work was supported by grants from the National Science Centre 2014/15/B/NZ3/00358 to AL.

## Supporting information


**Data S1:** Figure 1A raw data 1.

**Data S2:** Figure 1A raw data 2.

**SUPPLEMENTARY FIGURE S1.** Structural model of the ACS7 homodimer predicted by AF2. (A) ACS7 structure coloured by pDLLT (model confidence score). Graph showing the root mean square deviation (RMSD) for the ACS7 homodimer. The RMSD measures the average change in the displacement of the selected atoms relative to the reference. This plot was generated from MD data. (B) Diagram showing the positional fluctuations of all amino acids for the ACS7 homodimer structural model based on MD data; RMSF is the root mean square fluctuation calculated for the trace Cɑ or side chain of a given residue (chain). (C) The RMSF values for the predictions using the AF2 missing loops are highlighted. MD simulation was performed for ACS7 synthase with missing loops added from the AF2 prediction.
**SUPPLEMENTARY Figure S2.** The XBAT32 structural model was obtained from AF2. The structure was labelled using pDLLT (model confidence score), with Zn ions shown as spheres. The position of Zn ions was predicted based on structural alignment with the 4auq structure deposited in pdb. The N‐terminal region was excluded due to a low pDLLT score (this region seems to be disordered). For further analysis, only the 1–386 residue region of XBAT32 was used. This region contains two domains: RING and ANK repeats. The structure of XBAT32 was prepared using the prepwizard tool, minimised using the Prime module in the OPLS4 force field and used for further analysis. (A) Structural model of XBAT32 E3 ligase with UBC10 (E2 ligase) and a covalently bound Ub molecule. Complex obtained from the best scoring models according to the Haddock scoring function and with the lowest RMSD value compared to the 4auq structure (Tajdel‐Zielinska et al., 2024). (B) Possible complex of ACC7‐XBAT32‐UBC10‐Ub, taking into account which K residues are potentially ubiquitinated. The structure was obtained from the best scoring models based on the Haddock scoring function. Residues G76 and C85 are highlighted and selected K residues are labelled and shown as red sticks. Possible mechanism of ACS7‐XBAT32‐UBC10‐Ub complex formation showing selected K residues are accessible for transfer of the Ub molecule from E2 (UBC10) to target protein ACS7, where Ub is covalently bound to UBC10 via G76 and C85.
**SUPPLEMENTARY Figure S3.** (A) Graph showing the root mean square deviation (RMSD) for the XBAT32‐UBC1‐Ub complex. The RMSD measures the average change in the displacement of the selected atoms relative to the reference. This plot was obtained from MD data. (B) Diagram showing the positional fluctuations of all amino acids for the XBAT32‐UBC1‐Ub structural model based on the MD data; RMSF is the root mean square fluctuation calculated for the trace Cɑ or side chain of a given residue (chain). K90 was found to be very flexible according to the RMSF value. (C) Superposition of the obtained XBAT32‐UBC10‐Ub clusters from the MD simulation. C85 and G76, which form a covalent bond between UBC10 and Ub, are marked. Zn ions are marked as blue spheres. (D) Possible conformation of side chains of amino acids (D117, L119 and K90) surrounding C85 and G76. Data obtained after clustering the MD results.
**SUPPLEMENTARY Figure S4.** Blots used in Figure 1A.
**SUPPLEMENTARY Figure S5.** Blots used in Figure 5.

Table S1.

Table S2.

## Data Availability

All data generated during this study are included in this published article and supplementary files.
