# Supplementary material for: The HECT ubiquitin‐protein ligases UPL1 and UPL2 are involved in degradation of Arabidopsis thaliana ACC synthase 7
Source: Physiol Plant. 2025 Jan 6;177(1):e70030. doi: 10.1111/ppl.70030 (PMC11701798; doi:10.1111/ppl.70030)
Supplement: Supplementary file 1 — Data S1: Figure 1A raw data 1. [file PPL-177-e70030-s002.zip › GST_ACS7_WT_upl1_upl2_blots and Ponceau.pptx]

## Slide 1
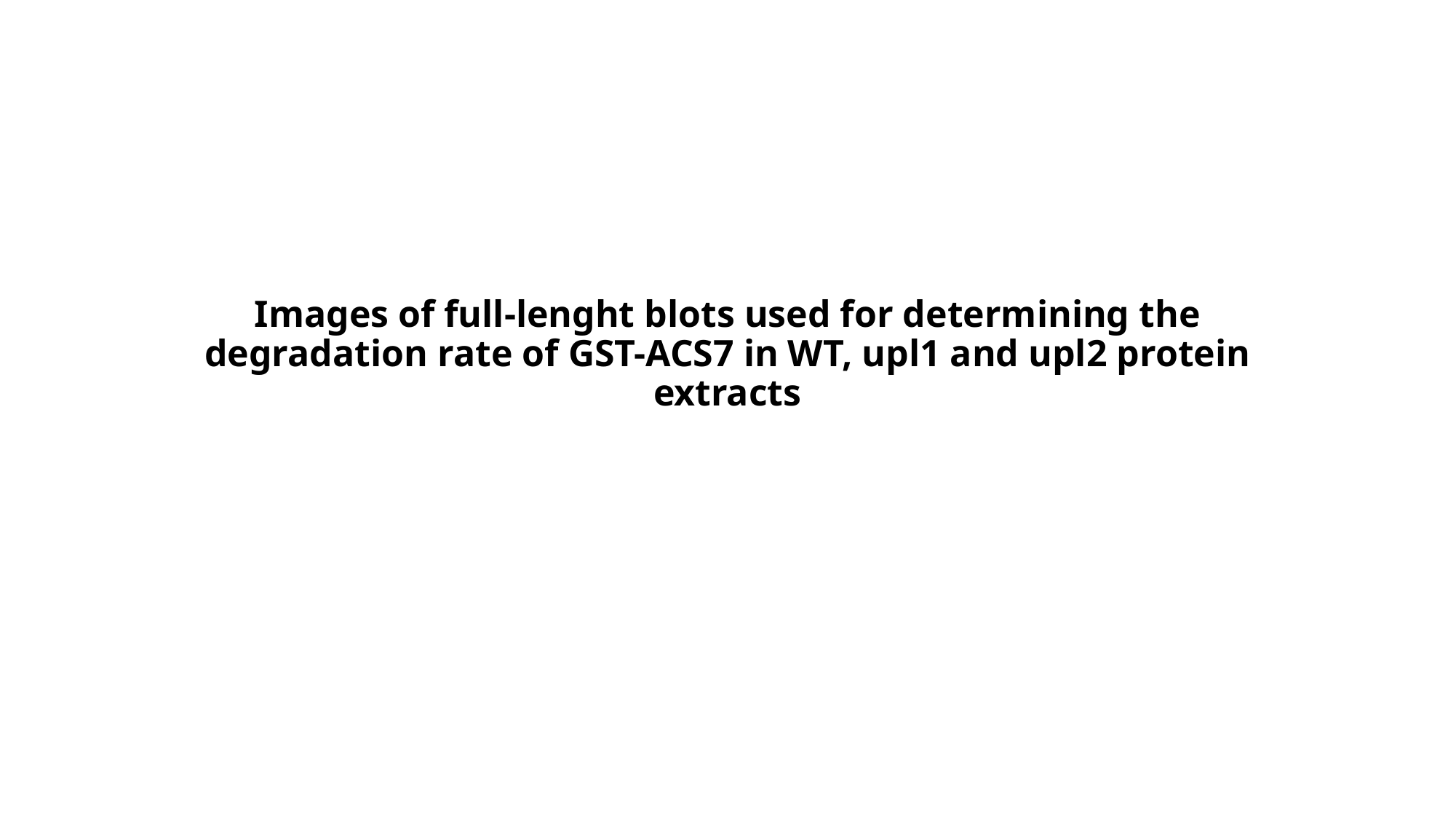

# Images of full-lenght blots used for determining the degradation rate of GST-ACS7 in WT, upl1 and upl2 protein extracts

## Slide 2
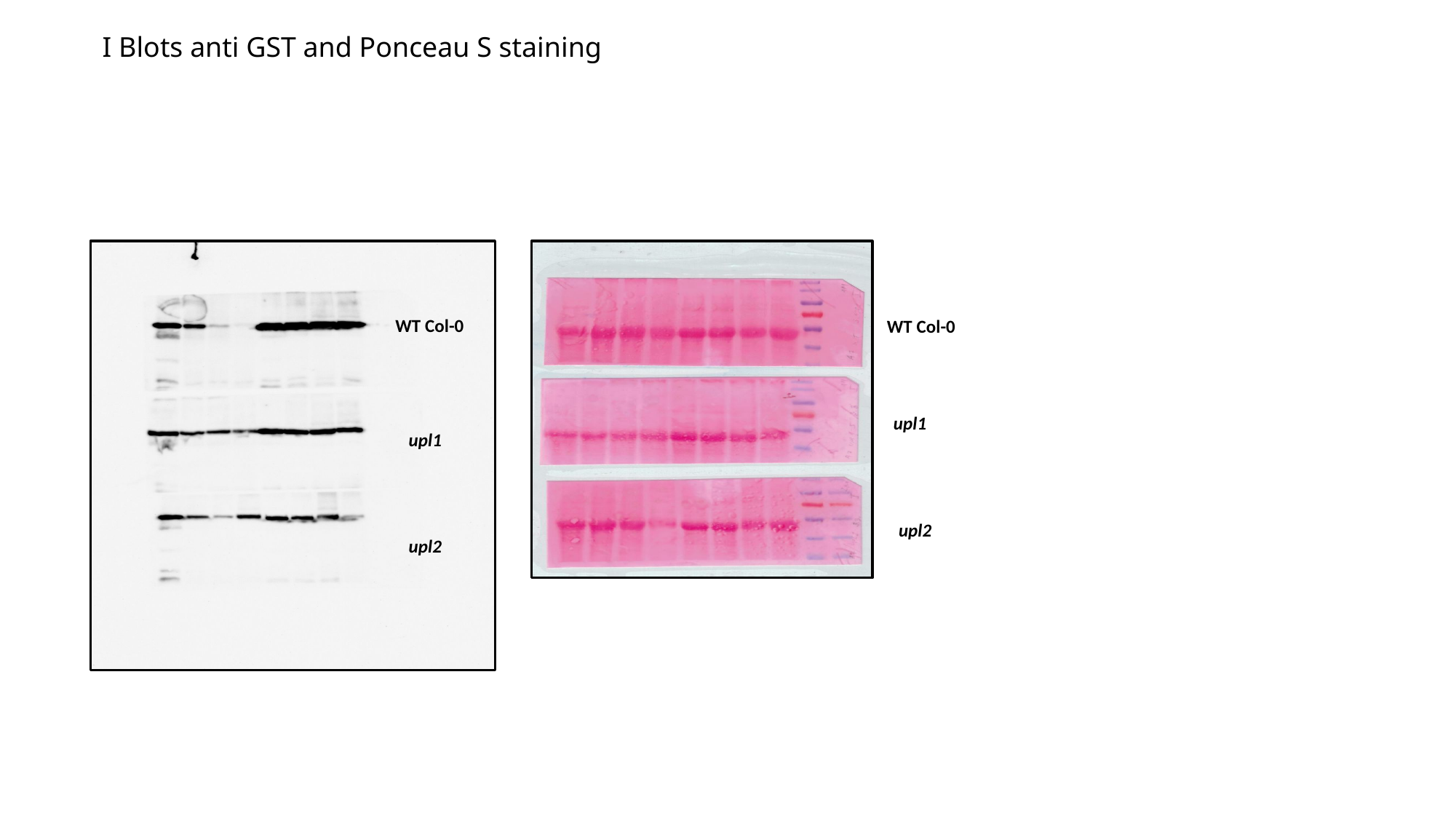

# I Blots anti GST and Ponceau S staining
WT Col-0
WT Col-0
upl1
upl1
upl2
upl2

## Slide 3
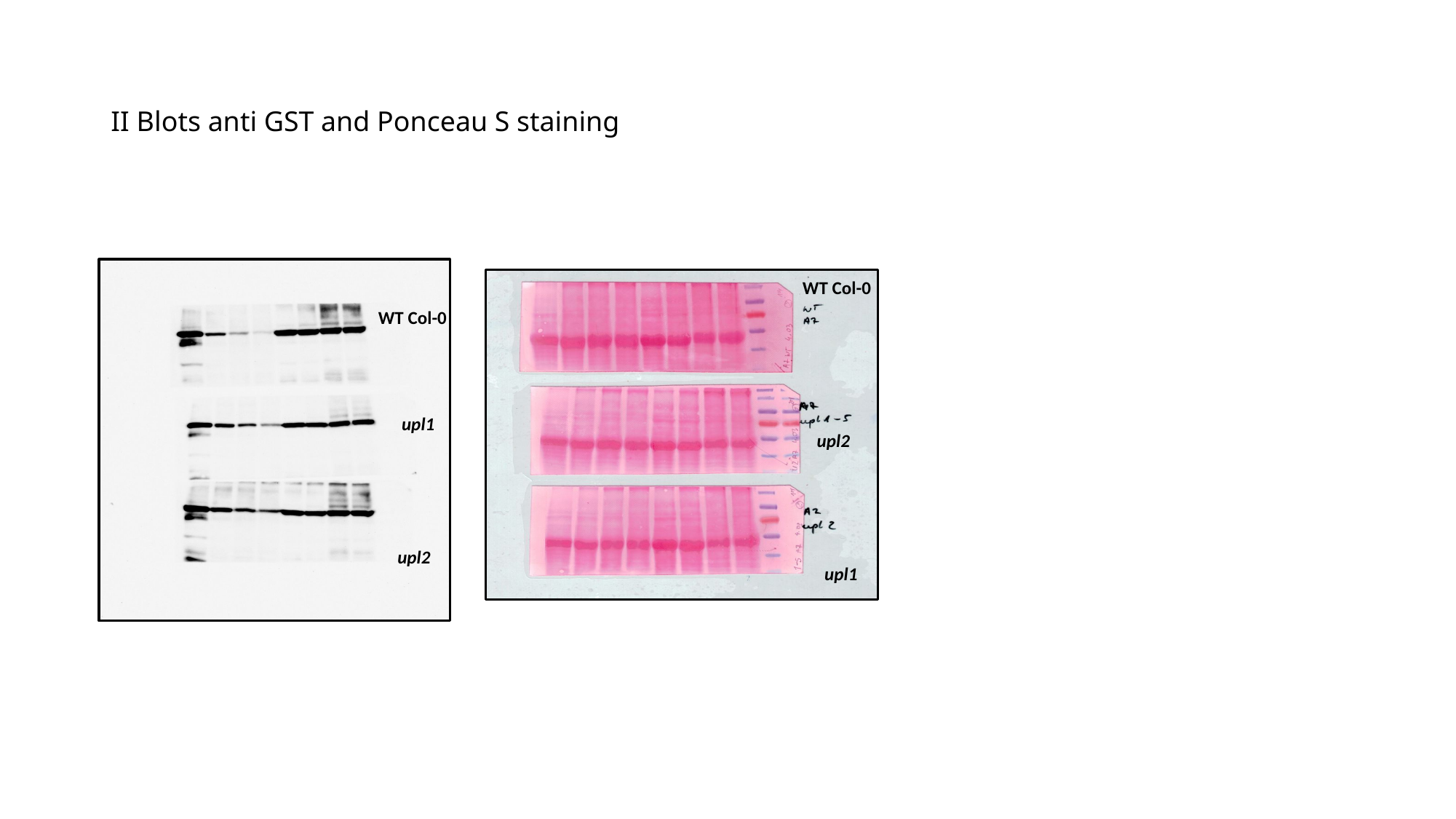

# II Blots anti GST and Ponceau S staining
WT Col-0
WT Col-0
upl1
upl2
upl2
upl1

## Slide 4
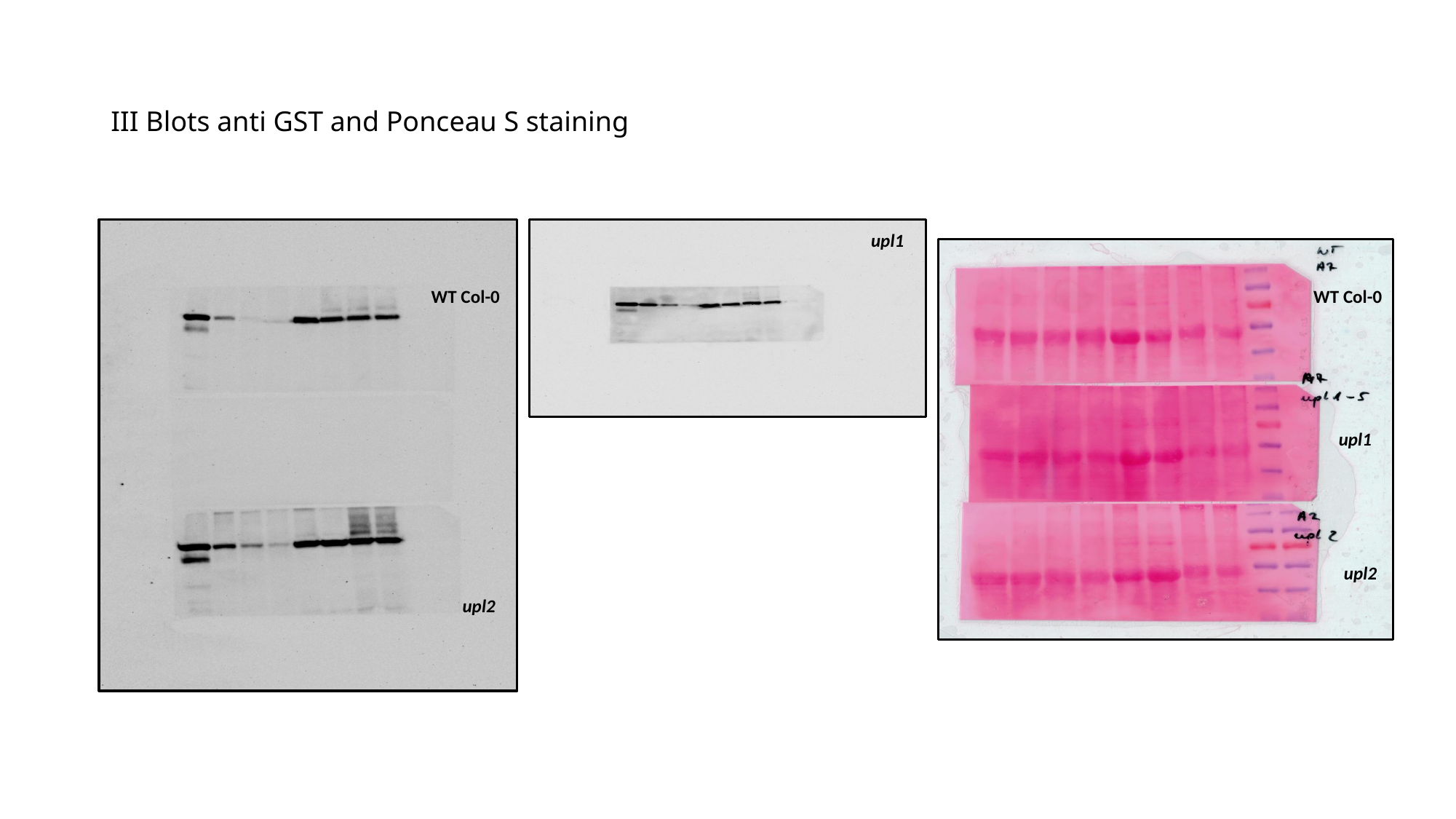

# III Blots anti GST and Ponceau S staining
upl1
WT Col-0
WT Col-0
upl1
upl2
upl2
