# Supplementary figures and images for: The HECT ubiquitin‐protein ligases UPL1 and UPL2 are involved in degradation of Arabidopsis thaliana ACC synthase 7
Source: Physiol Plant. 2025 Jan 6;177(1):e70030. doi: 10.1111/ppl.70030 (PMC11701798; doi:10.1111/ppl.70030)

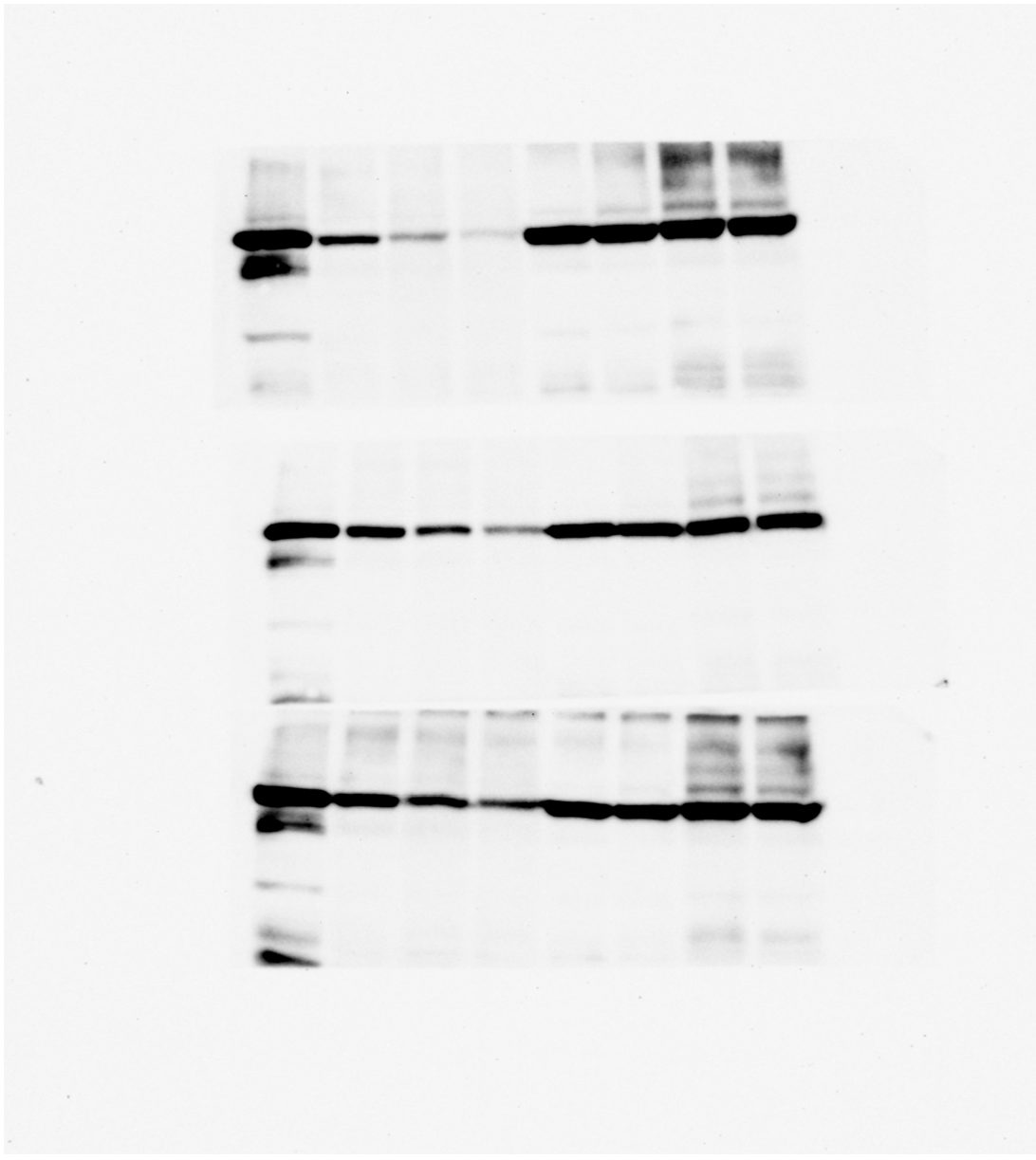

Supplement: Supplementary file 1 — Data S1: Figure 1A raw data 1. [file PPL-177-e70030-s002.zip › raw data GST-ACS7_WT_upl1_upl2/GST-ACS7 WT_upl1_upl2_II.tif]

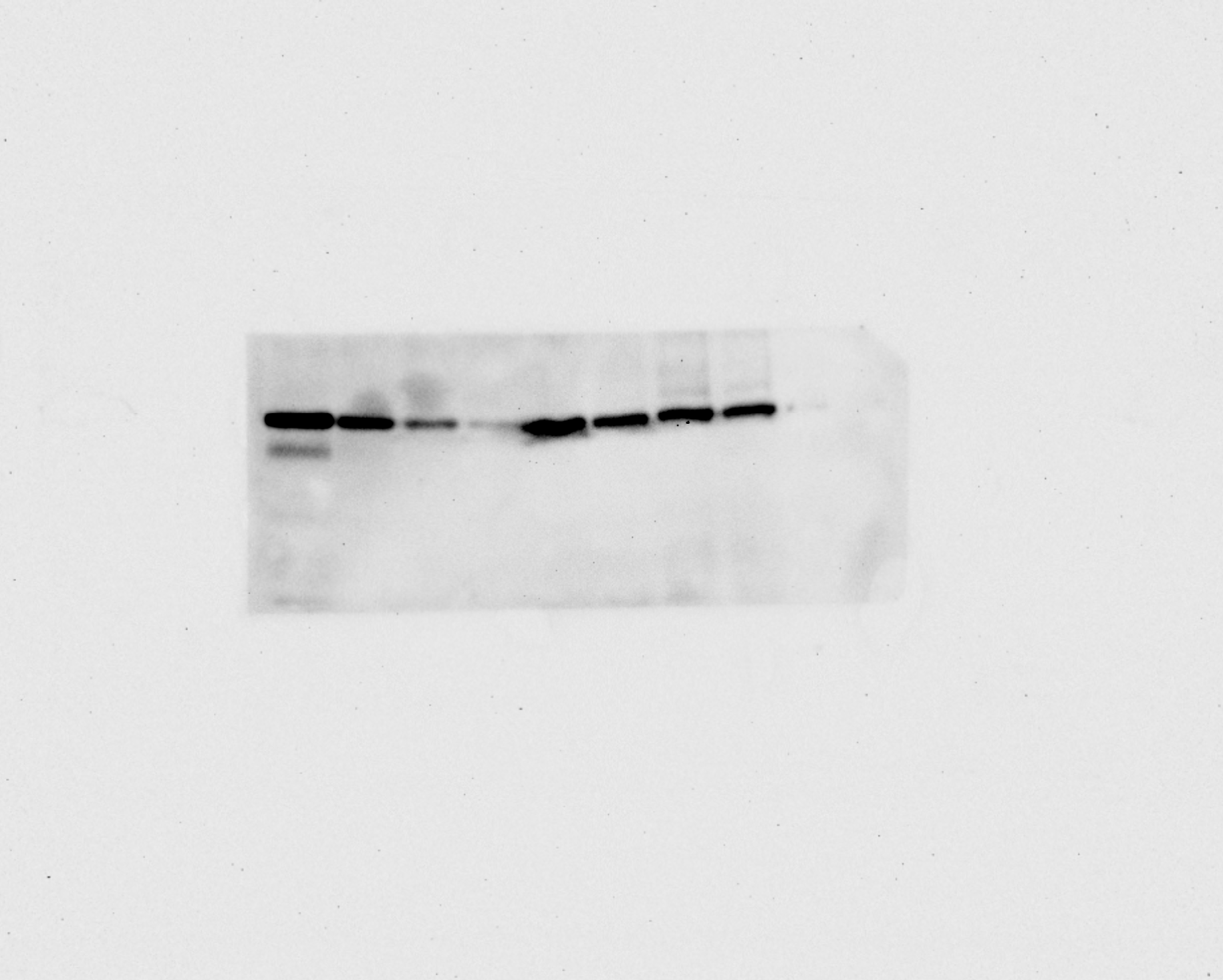

Supplement: Supplementary file 1 — Data S1: Figure 1A raw data 1. [file PPL-177-e70030-s002.zip › raw data GST-ACS7_WT_upl1_upl2/GST-ACS7_ upl_III.tif]

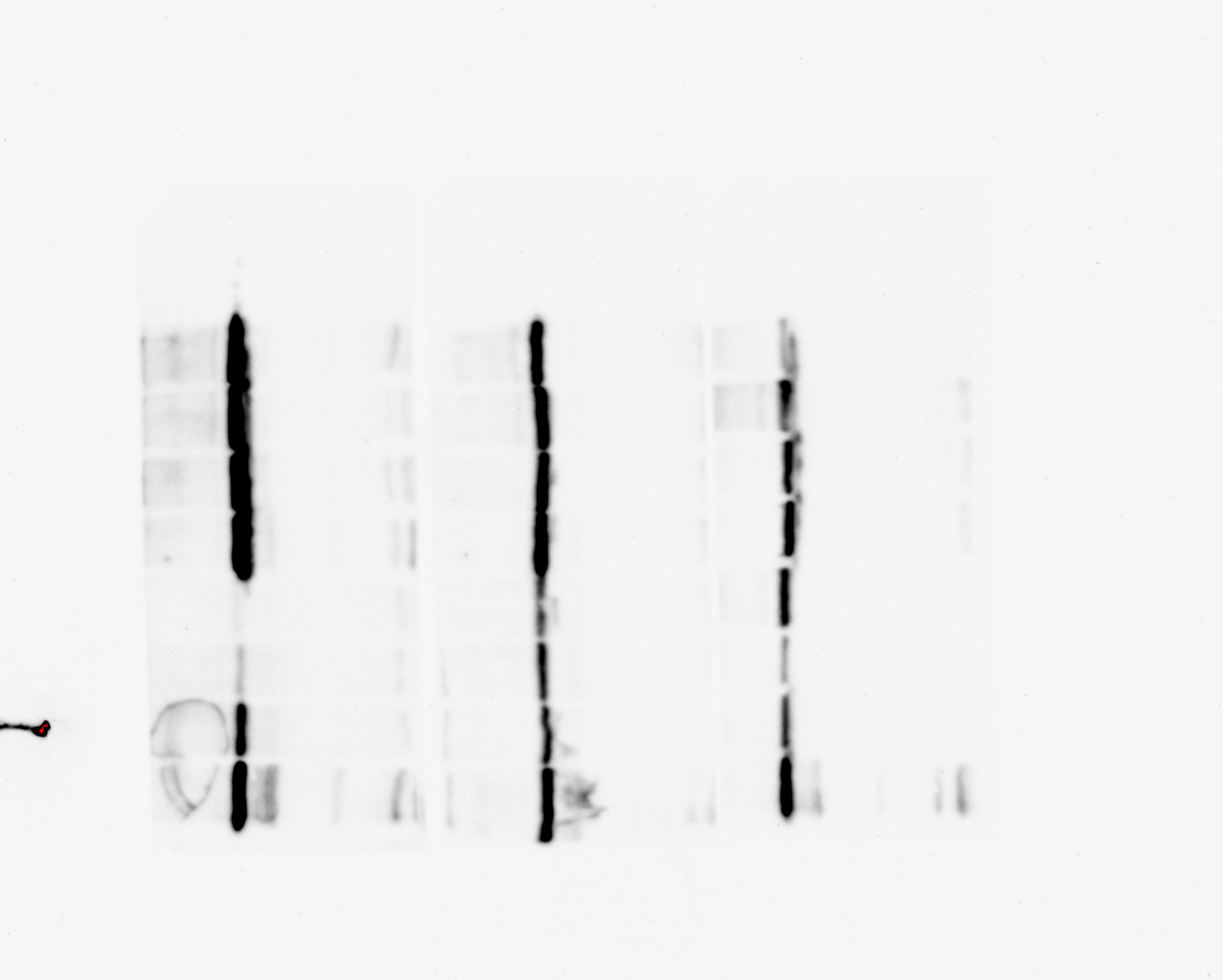

Supplement: Supplementary file 1 — Data S1: Figure 1A raw data 1. [file PPL-177-e70030-s002.zip › raw data GST-ACS7_WT_upl1_upl2/GST-ACS7_ wt_upl1_ upl2_I.tif]

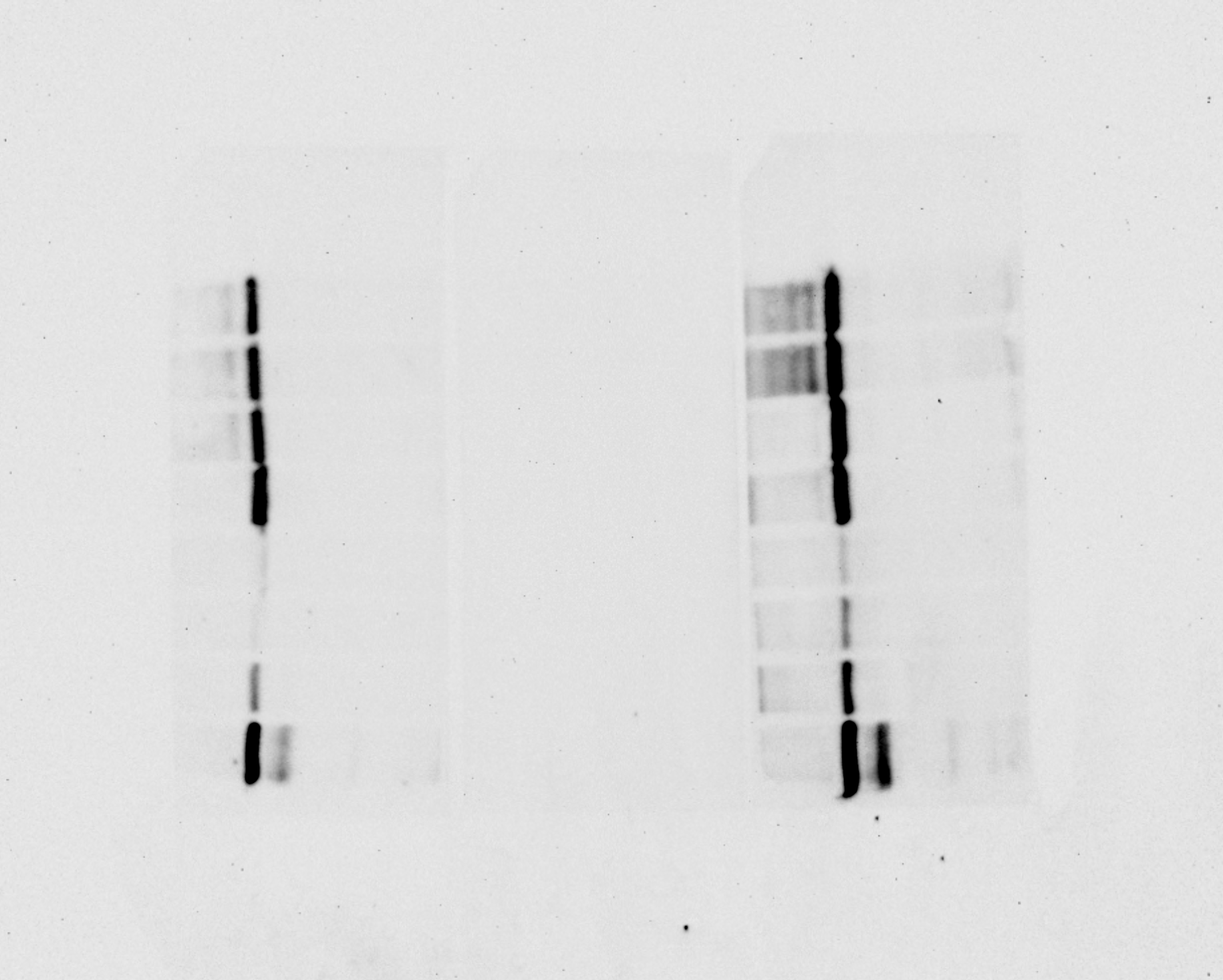

Supplement: Supplementary file 1 — Data S1: Figure 1A raw data 1. [file PPL-177-e70030-s002.zip › raw data GST-ACS7_WT_upl1_upl2/GST-ACS7_ wt_upl2_III.tif]

## Slide 1
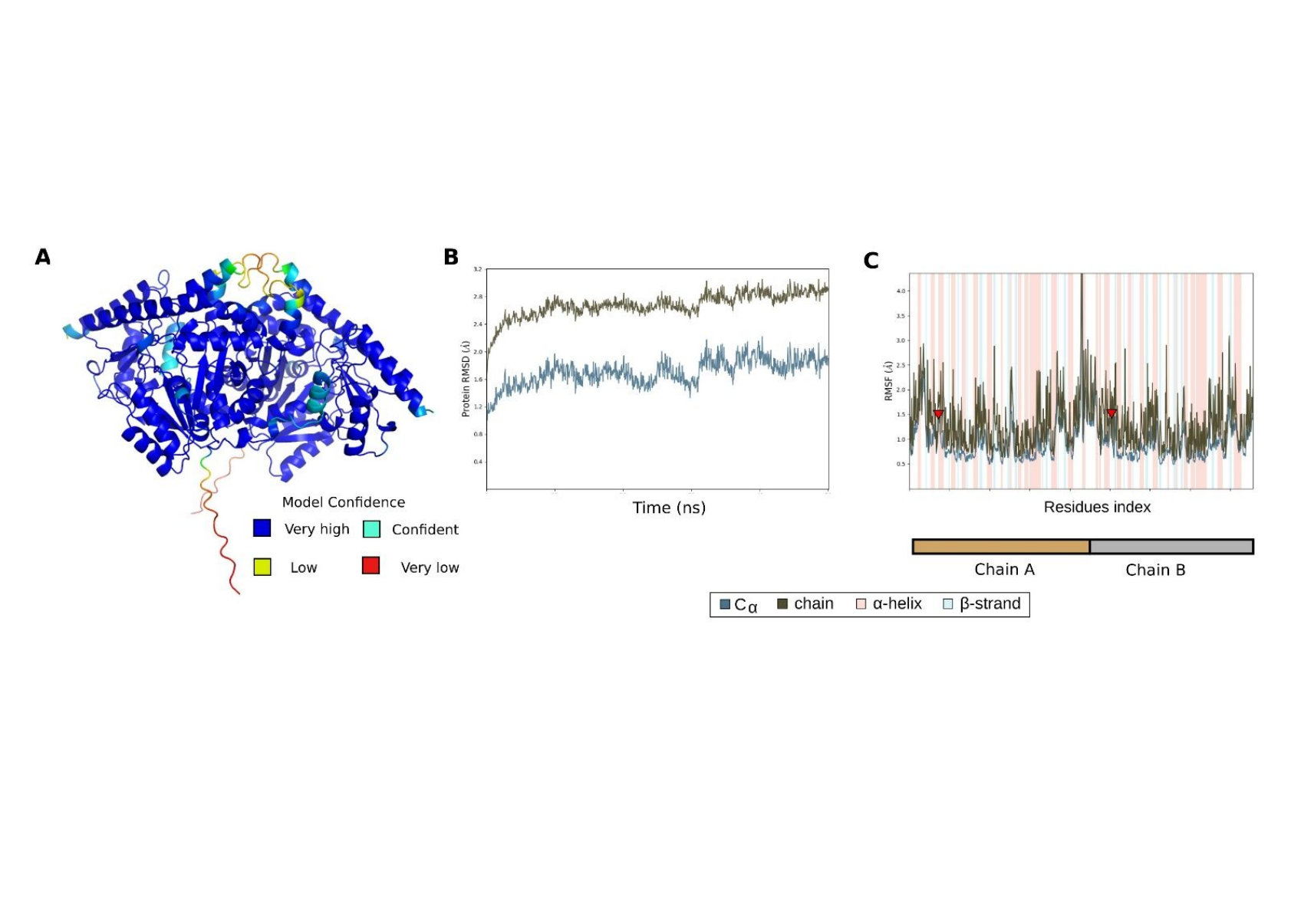

## Slide 2
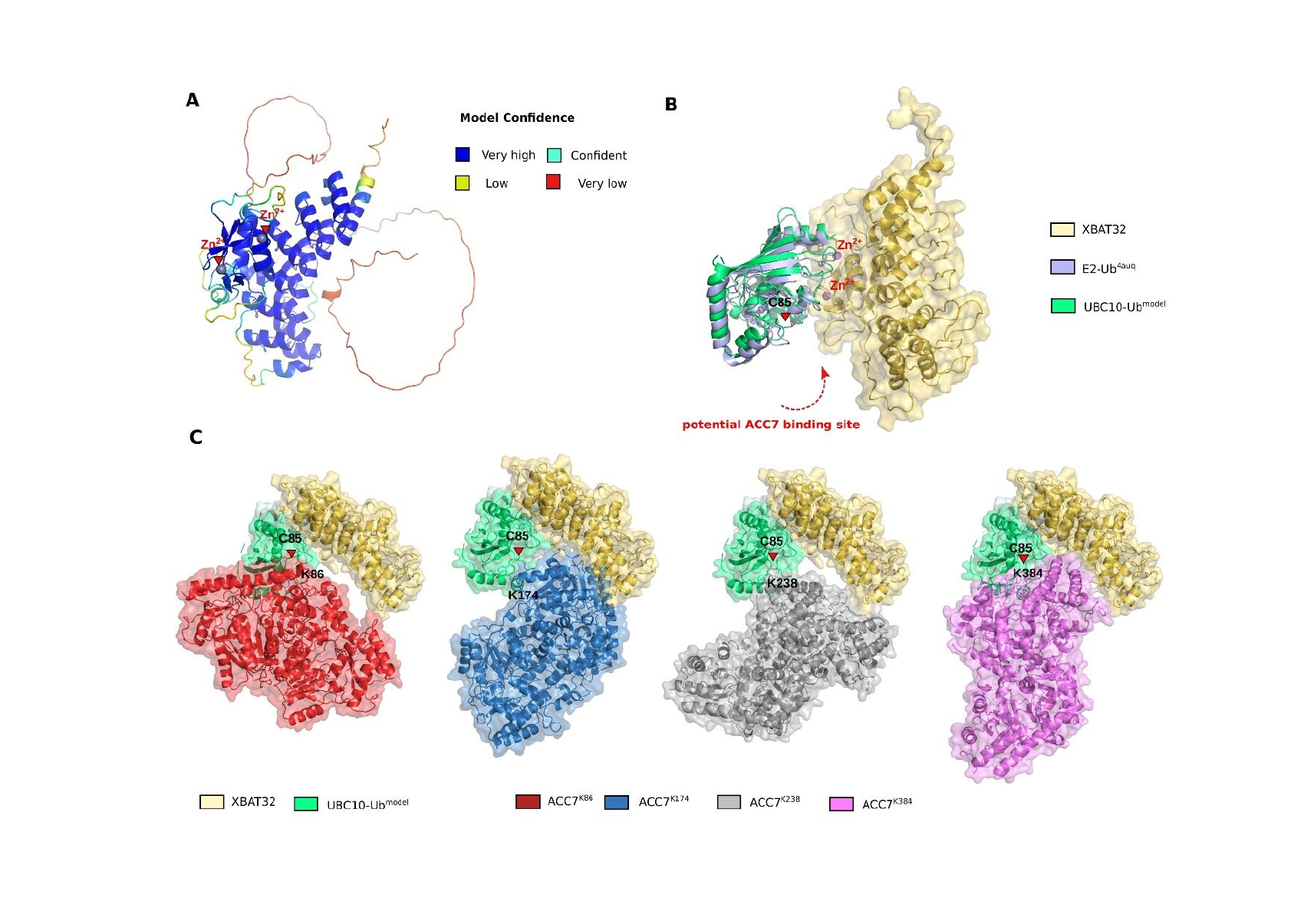

## Slide 3
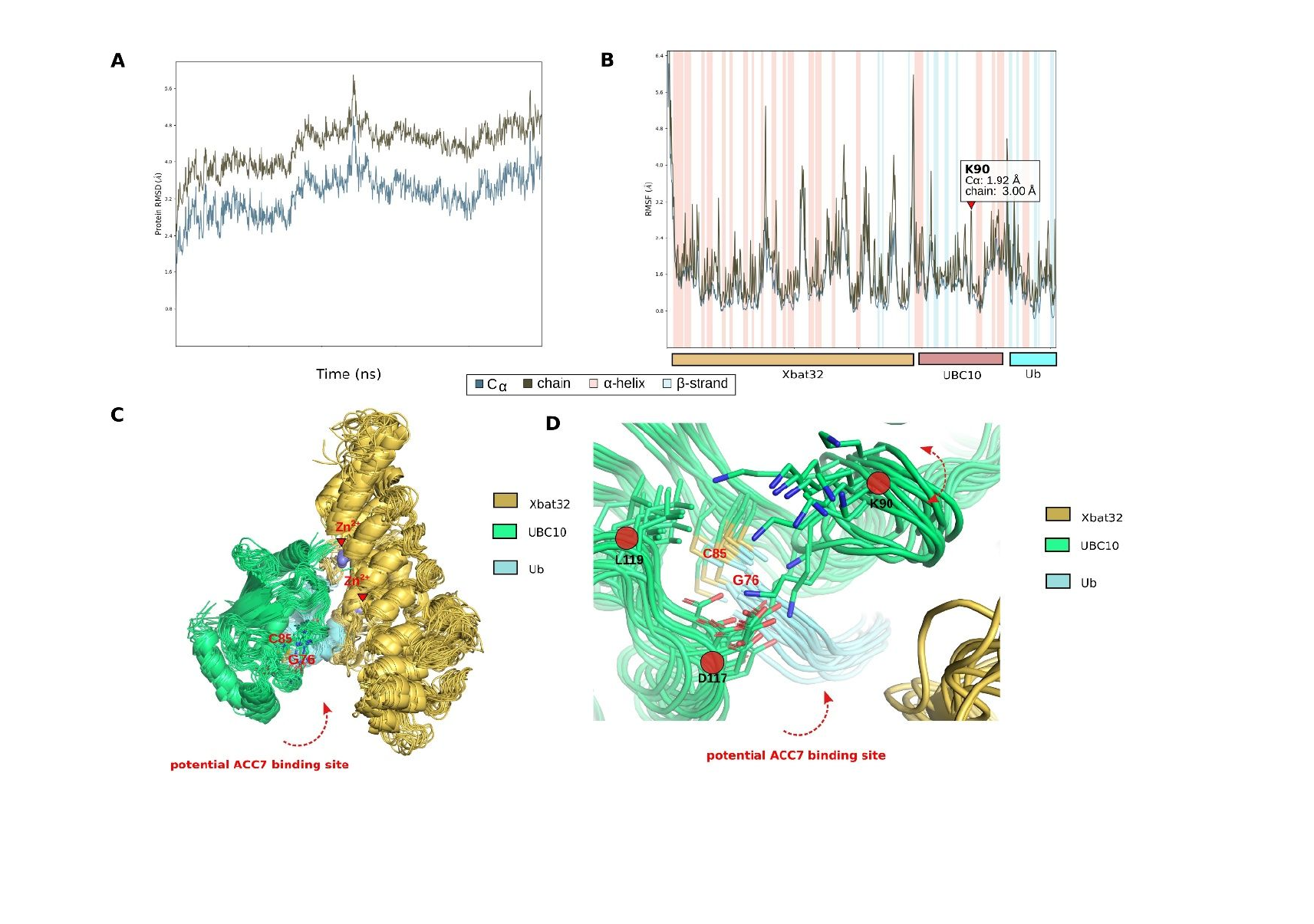

Supplement: Supplementary file 3 — SUPPLEMENTARY FIGURE S1. Structural model of the ACS7 homodimer predicted by AF2. (A) ACS7 structure coloured by pDLLT (model confidence score). Graph showing the root mean square deviation (RMSD) for the ACS7 homodimer. The RMSD measures the average change in the displacement of the selected atoms relative to the reference. This plot was generated from MD data. (B) Diagram showing the positional fluctuations of all amino acids for the ACS7 homodimer structural model based on MD data; RMSF is the root mean square fluctuation calculated for the trace Cɑ or side chain of a given residue (chain). (C) The RMSF values for the predictions using the AF2 missing loops are highlighted. MD simulation was performed for ACS7 synthase with missing loops added from the AF2 prediction. SUPPLEMENTARY Figure S2. The XBAT32 structural model was obtained from AF2. The structure was labelled using pDLLT (model confidence score), with Zn ions shown as spheres. The position of Zn ions was predicted based on structural alignment with the 4auq structure deposited in pdb. The N‐terminal region was excluded due to a low pDLLT score (this region seems to be disordered). For further analysis, only the 1–386 residue region of XBAT32 was used. This region contains two domains: RING and ANK repeats. The structure of XBAT32 was prepared using the prepwizard tool, minimised using the Prime module in the OPLS4 force field and used for further analysis. (A) Structural model of XBAT32 E3 ligase with UBC10 (E2 ligase) and a covalently bound Ub molecule. Complex obtained from the best scoring models according to the Haddock scoring function and with the lowest RMSD value compared to the 4auq structure (Tajdel‐Zielinska et al., 2024). (B) Possible complex of ACC7‐XBAT32‐UBC10‐Ub, taking into account which K residues are potentially ubiquitinated. The structure was obtained from the best scoring models based on the Haddock scoring function. Residues G76 and C85 are highlighted and selected K residues are [file PPL-177-e70030-s003.pptx]
